# *LigSearch*: a knowledge-based web server to identify likely ligands for a protein target

**DOI:** 10.1107/S0907444913022294

**Published:** 2013-11-19

**Authors:** Tjaart A. P. de Beer, Roman A. Laskowski, Mark-Eugene Duban, A. W. Edith Chan, Wayne F. Anderson, Janet M. Thornton

**Affiliations:** a European Molecular Biology Laboratory, European Bioinformatics Institute (EMBL–EBI), Wellcome Trust Genome Campus, Hinxton, Cambridge CB10 1SD, England; b Center for Structural Genomics of Infectious Diseases, Northwestern University Feinberg School of Medicine, Chicago, Illinois, USA; c Biological and Medicinal Chemistry Group, Wolfson Institute for Biomedical Research, University College London, London WC1E 6BT, England

**Keywords:** *LigSearch*, ligand prediction

## Abstract

Identifying which ligands might bind to a protein before crystallization trials could provide a significant saving in time and resources. *LigSearch*, a web server aimed at predicting ligands that might bind to and stabilize a given protein, has been developed. Using a protein sequence and/or structure, the system searches against a variety of databases, combining available knowledge, and provides a clustered and ranked output of possible ligands. *LigSearch* can be accessed at http://www.ebi.ac.uk/thornton-srv/databases/LigSearch.

## Abstract 2

*LigSearch* is a web server for identifying ligands likely to bind to a given protein. It can be accessed at http://www.ebi.ac.uk/thornton-srv/databases/LigSearch.

## 1. Introduction

Over the last few years a number of public small-molecule databases have been established, each with its own focus. The main databases are KEGG (Kotera *et al.*, 2012 ▶), BRENDA (Scheer *et al.*, 2011 ▶), ChEMBL (Gaulton *et al.*, 2012 ▶), ChEBI (de Matos *et al.*, 2012 ▶), ZINC (Irwin *et al.*, 2012 ▶) and PubChem (Bolton *et al.*, 2008 ▶). The KEGG small-molecule database focuses on substrates and products found in metabolic pathways and contains about 17 000 molecules. BRENDA is a collection of enzyme functional data and, as such, contains information about the small molecules (∼175 000) involved in enzymatic reactions. In comparison, the ChEMBL database contains ∼1.4 million bioactive, drug-like small molecules as well as data relating to various molecular properties such as logP and Lipinski parameters. ChEBI is an ontology-based dictionary of ∼34 000 biologically interesting small molecules. ZINC complements these databases by providing a free database of ∼21 million commercially available small molecules. The PubChem database contains information on the biological activities of small molecules as derived from the various NIH databases and contains data on over 100 million substances. Finally, the Worldwide Protein Data Bank (wwPDB; Berman *et al.*, 2003 ▶) holds over 64 000 three-dimensional structures of protein–ligand complexes and hence is an especially rich source of information on the binding of small molecules to proteins.

Given that a protein–ligand complex can provide valuable data on both the binding site of a protein and its biochemical function, crystallographers often need to identify small molecules that might bind to their protein. Rather than use trial and error, which is expensive and time-consuming, it is far better to identify potential ligands prior to the start of the experiments. However, even identifying likely molecules can involve a great deal of time and effort. For this reason, we have developed *LigSearch*, a web server that automates the process of identifying potential ligands for a given protein. The method uses sequence information and so is suitable for proteins of known and unknown three-dimensional structure alike.

It should be noted that the aim of *LigSearch* is not to identify a protein’s binding site, although in some cases this may be a spin-off. There are plenty of methods that do this already, some using structural information, some using sequence information and others using a combination of both. The server merely aims to use various existing resources to identify small molecules that are likely to bind to a given protein. It then clusters the results, grouping the molecules by their similarity, and ranks the clusters and the molecules within each cluster using a scoring scheme that aims to place the more promising hits nearer the top of the output.

## 2. Methods

**Figure 1:**
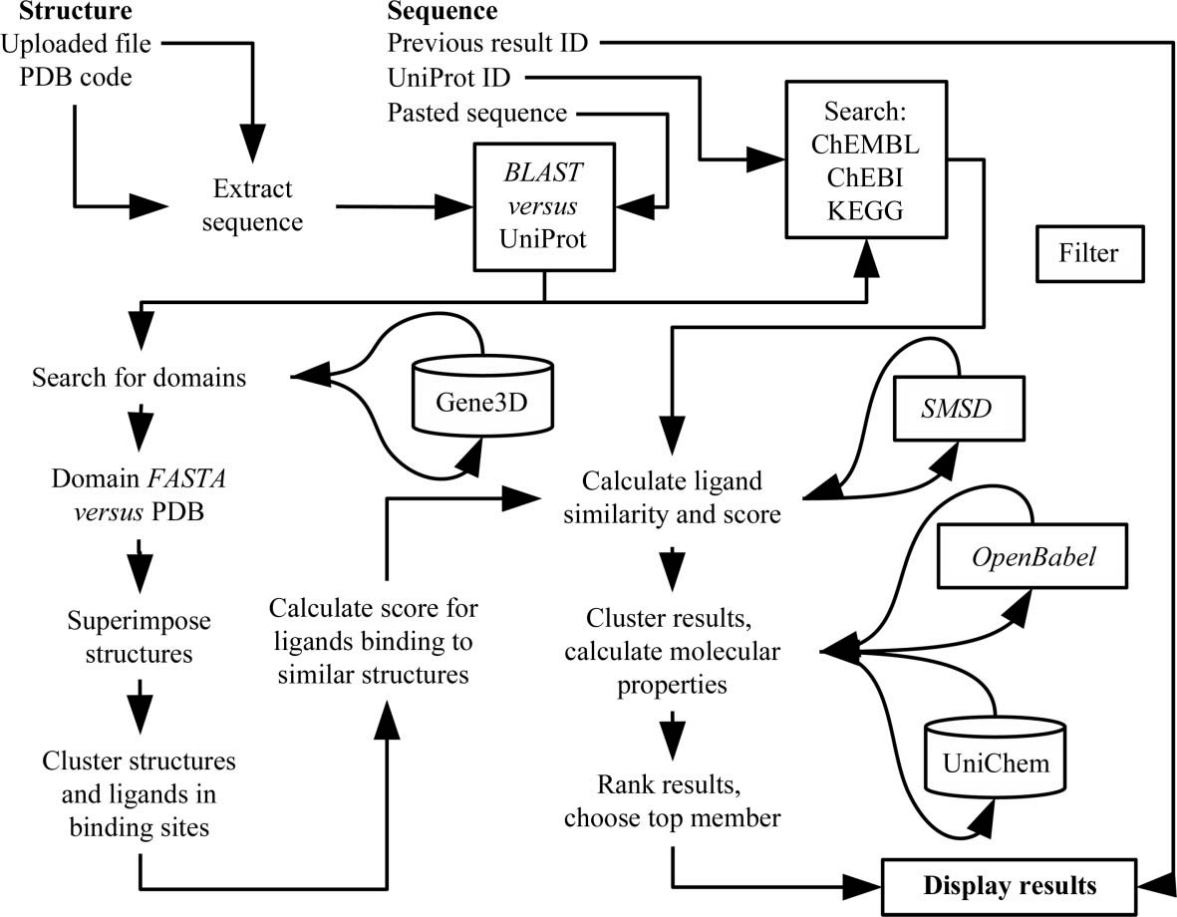
A schematic outline of the workflow in *LigSearc*h. The user can submit either a structure or a sequence.

The *LigSearch* pipeline is shown schematically in Fig. 1 ▶ and is described in more detail below. The user can submit a protein sequence (either *via* a UniProt ID or a pasted sequence) or a protein structure (*via* a PDB code or an uploaded PDB file), from which the sequence is extracted. Results are emailed to the user in the form of a password-protected link to a web page of ranked ligand hits.

### 2.1. Sequence searches

The submitted protein sequence is first searched against the curated entries in UniProtKB/Swiss-Prot (The UniProt Consortium, 2012 ▶) using *BLAST* (Altschul *et al.*, 1990 ▶). The top 20 sequences matched represent the query sequence plus a set of its closest relatives. The UniProt identifiers for each of these 20 hits are searched against ChEMBL, ChEBI and KEGG, using their respective web services, to retrieve the small-molecule compounds that these databases have associated with each of the given proteins. For the most part, these data come from the scientific literature. ChEMBL search results are filtered to remove compounds having no binding constants and those with an IC_50_ value of more than 100 n*M*. ChEBI search results are filtered to exclude all compounds with fewer than four atoms and/or a molecular weight of less than 50 Da. The results from the three searches are combined and duplicates are removed, giving a list of compounds known to be associated with the query sequence and/or its closest relatives.

### 2.2. Searches against the PDB

The original sequence is then searched against the protein sequences in the PDB. To improve the chances of successful matches, and to increase the number of hits, the sequence is first broken up into regions that are likely to correspond to structural or sequence domains. This is performed as follows. The top 20 *BLAST* hits found above are located in the Gene3D database (Lees *et al.*, 2012 ▶), which primarily identifies likely structural domains in 15 million UniProt sequences. Gene3D is compiled by deriving hidden Markov models (HMMs) to map sequences to the protein structural domains defined in the CATH domain database (Sillitoe *et al.*, 2013 ▶). Any long stretch of sequence that cannot be mapped to a CATH domain is instead mapped to a Pfam (Finn *et al.*, 2010 ▶) sequence domain.

From the Gene3D domain assignments for the 20 UniProt sequences, the query sequence is partitioned into predicted domains. Starting with the most similar sequence, the domains are applied to the query sequence provided that they do not overlap with a previously assigned domain. Once the domains have been assigned, any unassigned regions of over 100 residues are designated domains of unknown type. Linker sections between domains are split in half and each half is assigned to the domain adjacent to it.

The sequence of each of these protein domains is then searched against the sequences in the PDB. Again, to increase the chances of successful matches, the database of sequences contains not just the full-length sequences of each protein chain in the PDB but also the sequences corresponding to each CATH domain. The latter are particularly important for matching ‘split’ domains, which might otherwise be missed by the sequence search. For example, PDB entry 1got consists of two CATH domains, the first being a domain comprising residues 6–57 and 177–331 and thus ‘split’ over two segments of the protein, while the second domain spans the region in between, residues 58–176.

The search itself is performed using *FASTA* (Lipman & Pearson, 1985 ▶) and a multiple alignment is derived from the resultant pairwise sequence alignments. For each domain searched, the structure with the best match (based upon the maximum Smith–Waterman score produced by *FASTA*) is used as a reference structure and all others are superposed onto it using the main-chain atoms of equivalent residues in the alignment. Several iterations of superposition may be required to obtain an r.m.s.d. below a 12 Å cutoff, with the residues having the highest r.m.s.d. values being removed at each iteration. This compensates for any imperfections in the sequence alignment.

The superposition of all of the structures onto the reference structure brings with it any bound ligands. The net result is that the various ligands overlap in one or more binding sites of the superposed proteins. These ligands represent the predicted binding partners of the query protein. A scoring system is used to order them from the most to the least promising candidates. The scores take into account the numbers and types of interaction that each ligand makes with its protein partner and also the similarity between the residues that it interacts with and the corresponding residues in the query protein. Specifically, the ligand scores 1 for each hydrogen bond it makes to the protein, with the score being 2 if the interaction is with a similar residue to that in the query sequence, 3 if it is with an identical residue type and −1 if there is no equivalent (*i.e* a gap in the alignment). The equivalent scores for nonbonded contacts are 0, 1, 2 and −1, respectively, although where there are several contacts to any given protein residue only one is counted.

The scoring system is somewhat arbitrary and is difficult to optimize without extensive experimental ligand-binding data; however, its aim is merely to provide a qualitative ranking of ligands according to how similar the residues they interact with are to the corresponding residues in the query sequence.

Many ligands in the PDB interact with more than one protein domain, so a domain-based sequence search and superposition will miss any interactions that the ligand makes with other domains. This is taken into account by merging the results from the separate domain searches.

### 2.3. Molecular similarity and result ranking

The ligands identified by the three sequence searches and those from the search against the PDB are clustered according to their molecular similarity as calculated by *SMSD* (*Small Molecule Subgraph Detector*; Rahman *et al.*, 2009 ▶). *SMSD* computes the maximum common subgraph between two small molecules and provides a similarity score based on the matching subgraphs. Clustering uses a similarity cutoff of 0.4 between the most distant members of the cluster and is solely based on molecular similarity and not on whether the molecules bind in the same protein binding site.

The clusters obtained are ranked on the basis of the highest interaction score for the PDB ligands in each cluster. Within each cluster the PDB ligands are ranked by their interaction scores, while the hits from ChEMBL, ChEBI and KEGG, having no interaction score, are listed at the end of each cluster in decreasing order of the sequence similarity of their associated protein to the query sequence and then by their number of cross-references in UniChem (Chambers *et al.*, 2013 ▶). UniChem is a nonredundant database of links between chemical structures and EMBL–EBI chemistry resources. The number of cross-references indicates in how many other databases the small molecule appears. This provides a qualitative measure of its likely ‘importance’.

*OpenBabel* (O’Boyle *et al.*, 2011 ▶) is used to calculate various molecular properties such as logP and the polar surface area where such data are lacking. The ordered list of predicted ligands can be downloaded in a tab-separated file.

### 2.4. Validation and benchmarking

To validate the *LigSearch* pipeline, we chose a set of enzymes as our test group of proteins. For the most part, the molecules that bind to enzymes are known (the reactants, products and any cofactors) and hence provide a means of validating the *LigSearch* predictions. However, because of the very fact that the cognate ligands are known, they will inevitably be returned by the KEGG, ChEBI and ChEMBL searches and so such hits need to be discarded before analysing what is left.

Furthermore, if the three-dimensional structure of any of the enzymes is known, this will bias the results returned from the searches against the PDB. To prevent this, we first used the Enzyme Structures Database, part of PDBsum (Laskowski, 2009 ▶), to select a data set of enzymes which have no three-dimensional structure in the PDB. This gave 3431 EC classes (as of July 2013). From these, we selected only those enzymes whose cognate molecules were given in the ENZYME database (Bairoch, 2000 ▶) and had an associated .mol file in the Enzyme Structures Database. This was to ensure that the correct answers (*i.e* the substrates and/or cofactors) were known and could be compared against the molecules returned by *LigSearch*. The result was a set of 2334 enzymes.

A list of proteins belonging to each of these enzyme classes was then extracted from UniProtKB/Swiss-Prot (*i.e.* the reviewed part of UniProtKB). The 2334 enzyme classes encompassed 195 754 UniProtKB sequences. To select a manageable data set, the sequences were randomly selected from this list, ensuring that no EC class was represented more than once, to give 200 proteins. The number of identifiable reaction molecules associated with this set was 620. The search sequences and reaction molecules are listed in Supplementary Table S1,^1^ together with the results of the searches, as described below.

**Figure 2:**
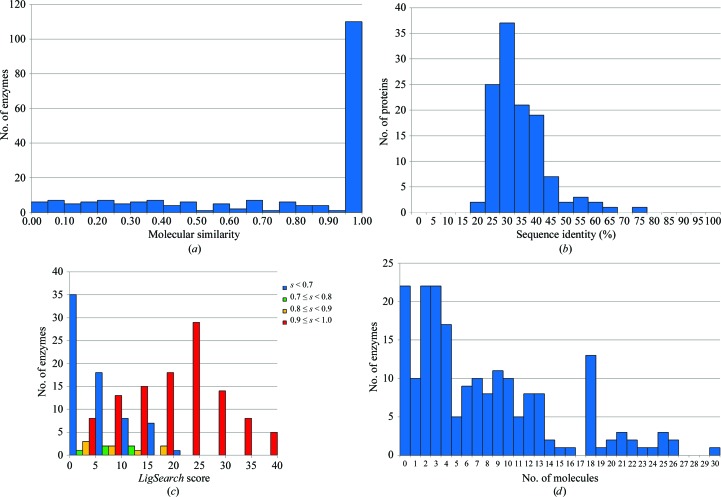
Validation results for *LigSearch* runs on 200 randomly selected enzymes with no three-dimensional structural model in the PDB. (*a*) Histogram of the molecular-similarity scores for the closest PDB ligand match, as computed by the *SMSD* program, to any of the enzyme’s cognate ligands. (*b*) Histogram of the sequence identities between the query enzyme sequence and the PDB protein from which the best ligand match has a similarity of 0.8 or greater to one of the cognate ligands. (*c*) Histogram of the *LigSearch* scores for the best matches to cognate ligands. The counts are grouped into four sets according to the similarity score, *s*, of the best-matching molecule. Lowest similarity scores (*s* < 0.7) are shown in blue, scores 0.7 ≤ *s* < 0.8 are shown in green, scores 0.8 ≤ *s* < 0.9 are shown in orange and closest matches with *s* ≥ 0.9 are shown in red. (*d*) Histogram of counts of molecules with similarity *s* ≥ 0.8 to at least one of the enzyme’s cognate ligands as returned by *LigSearch* for the non-PDB hits. The cognate molecules themselves are, of course, excluded from the results.

Firstly, we analyzed the results returned by the searches against the PDB. For each of the 200 protein sequences, the PDB ligands returned by *LigSearch* were compared against the protein’s cognate molecules using the *SMSD* program. For 102 (51%) of the proteins at least one of the molecules suggested by *LigSearch* was a perfect match to one of these cognate molecules. In a further 18 cases a molecule with a match score of 0.8 or higher to a known substrate was identified. Thus, for 120 of the 200 enzymes (60%) an identical or very similar molecule to a known binder was identified in the PDB (see Fig. 2 ▶ *a*). These were not merely trivial matches from close homologues. Just two of the 200 came from a protein with sequence identity higher than 65% to the query sequence. Over 50% came from proteins with a sequence identity of 30% or less (see Fig. 2 ▶ *b*).

As described above, the *LigSearch* score for each PDB hit reflects the similarity between the residues interacting with the ligand in the PDB complex and the corresponding residues in the query protein. The higher the score, the more equivalent interactions with identical or similar residue types are possible. Indeed, the best matches in the enzyme data set tend to have the highest *LigSearch* scores (Fig. 2 ▶ *c*), so the scores do provide a guide to which molecules are more likely to bind to the query protein. Indeed, from the results in Fig. 2 ▶(*c*) it would appear that ligand scores higher than around 10–15 tend to be associated with the correct answers.

Secondly, to test the results returned by the ChEBI, ChEMBL and KEGG searches, we took each cognate molecule in turn and counted how many other molecules were returned in the same cluster as the cognate molecule. Fig. 2 ▶(*d*) shows the results. For 22 (11%) of the enzymes none of the molecules returned by the ChEBI, ChEMBL and KEGG searches were similar enough to any of the cognate molecules to be in the same cluster. However, for the remaining 178 (89%) of the enzymes at least one of the small molecules returned was similar to one of the cognate ligands.

Together, the validation study suggests that in the majority of cases the answers returned by *LigSearch* include molecules that are highly likely to bind, owing to their high similarity to known binders, and these molecules tend to be those that score highly using the *LigSearch* interaction score.

## 3. Results

To demonstrate the usefulness of the system in practice, we obtained ligand-testing data from one of the crystallo­graphers at the Midwest Center for Structural Genomics (MCSG). Her project had necessitated a search for candidate molecules to cocrystallize with thymidylate synthase from *Staphylococcus aureus* (UniProt ID P65248). Using results from literature mining, she had identified a number of potential compounds in February 2012. Of these, 26 were selected and 13 were used in crystallization trials (E. Filippova, personal communication). Table 1 ▶ lists the 26 compounds. The structures of two protein–ligand complexes were eventually solved and deposited in the PDB as entries 4dwj and 4eaq in February and March 2012, respectively. In fact, 4dwj was a trivial case as the ligand selected had already been solved in complex with the same protein (PDB entries 2ccg and 2ccj).

We submitted this protein sequence to *LigSearch* to compare the hits returned against the molecules that had been manually compiled. The PDB contains many thymidylate synthase structures from various organisms, so it was not surprising that *LigSearch* returned many hits. All structures solved after February 2012 were discarded in order to present the results as they would have been at the time of the original study. In all, 62 unique ligands were matched in the PDB. Additionally, a further 126 unique molecules were obtained from ChEBI. In this example, no hits were returned by the ChEMBL and KEGG searches.

**Figure 3:**
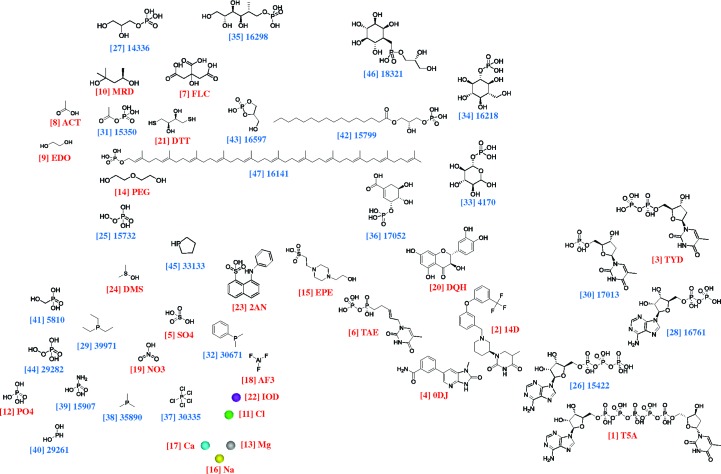
A plot of the top-scoring molecules in each of the 47 clusters returned by *LigSearch* for UniProt entry P65248. The molecules have been laid out using multi-dimensional scaling on the basis of their all-by-all similarities. Thus, similar molecules tend to be grouped together. The labels show the cluster number in square brackets and the PDB Het Group three-character name or ChEBI identifier. Red labels correspond to molecules from matches to PDB entries, while blue labels are molecules returned by ChEBI searches. The molecular diagrams were plotted using *ChemDraw* (http://www.cambridgesoft.com).

The clustering of all of the candidate molecules by *SMSD* resulted in 47 separate clusters, five of which contained a single metal ion. Fig. 3 ▶ shows the highest-scoring members of each of these 47 clusters plotted using multi-dimensional scaling on the basis of their all-by-all similarities.

The top-scoring clusters, ranked 1, 2 *etc.*, tend to group in the bottom right-hand corner of the plot. The metals and various very small molecules are grouped at the bottom left.

**Figure 4:**
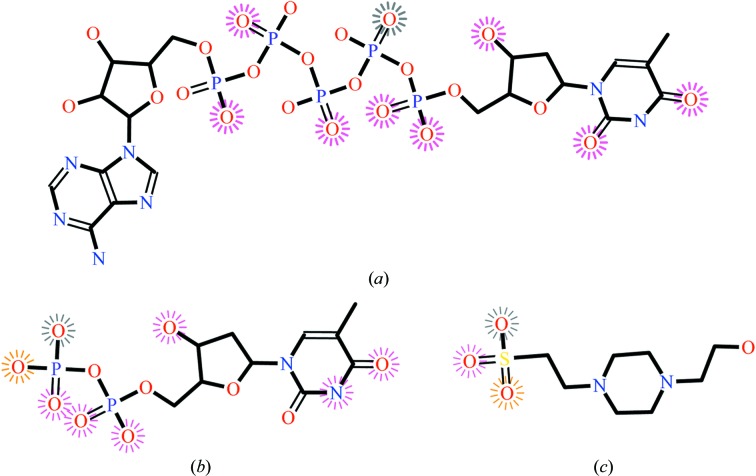
Three cluster representatives for the molecules listed in Table 1 ▶. The molecules are annotated according to the interactions that they make with the protein in the top-scoring PDB entry for the cluster. Atoms making hydrogen bonds to protein are depicted with spokes radiating from them, while hydrophobic interactions have a grey circle around them (none in this example). The colour of the spokes corresponds to the similarity of the residue to which the hydrogen bond is made and the corresponding residue in the query protein (which in this case is thymidylate synthase from *S. aureus*; UniProt ID P65248): red for identical residue type, orange for similar and dark grey for different. The images are provided in the results section for every query with PDB hits. The molecules and the PDB entries from which the data come are (*a*) *P* ^1^-(5′-adenosyl)-*P* ^5^-(5′-thymidyl)pentaphosphate (PDB entry 4tmk), (*b*) thymidine 5′-diphosphate (PDB entry 3hjn) and (*c*) 4-(2-hydroxyethyl)-1-piperazineethanesulfonic acid (PDB entry 2pir).

Most of the 26 compounds from the manual selection exercise listed in Table 1 ▶ were identified in the *LigSearch* output and indeed fell into three of *LigSearch*’s clusters, 1, 3 and 15, as shown in the table. The top-scoring ligand in each of these three clusters is depicted in Fig. 4 ▶, showing the atoms that interact with the protein. In fact, *LigSearch* identified even higher scoring molecules than those that had been selected by hand, but these are not shown in the table. *LigSearch* missed two of the 26 manually selected compounds, but in both cases the compounds are substructures of other molecules returned by *LigSearch* and thus in effect are not significant omissions.

The rightmost columns of the table show the PDB entries from which the ligands came and the sequence similarity of each protein to the query sequence. Many of the latter lie in the 20–30% range, suggesting the predicted ligands come from distantly related proteins. Their high interaction scores, however, are suggestive of conservation in the binding site and indicate that there is a strong chance the ligands may bind to the query protein.

**Figure 5:**
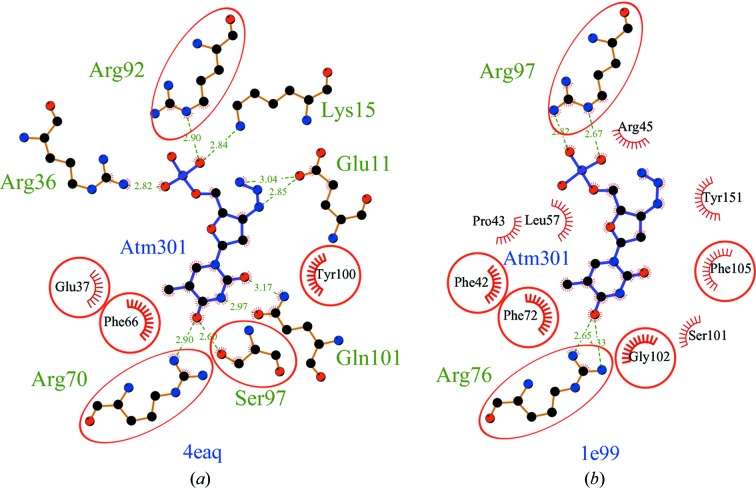
A schematic diagram of the protein–ligand interactions in two distantly related proteins: (*a*) thymidylate synthase from *S. aureus* (PDB entry 4eaq) and (*b*) human thymidylate synthase (PDB entry 1e99). The ligands (blue bonds) in both are identical: 3′-azido-3′-deoxythymidine 5′-­monophosphate. Equivalent protein residues in the two plots are circled in red and occupy the same positions in each plot: for example, Glu37 is equivalent to Phe42, Phe66 is equivalent to Phe72 and Tyr100 is equivalent to Phe105. Hydrogen bonds are depicted by green dotted lines and labelled with their length in Å, while hydrophobic interactions are represented by red arcs whose spokes radiate towards the ligand. The diagram was generated using *LigPlot* ^+^ (Laskowski & Swindells, 2011 ▶).

Fig. 5 ▶ shows an example of such a case. It compares the protein–ligand interactions for the same 3′-azido-3′-deoxy­thymidine 5′-monophosphate ligand bound to *S. aureus* thymidylate synthase in PDB entry 4eaq and human thymidyl­ate synthase in PDB entry 1e99. Despite the low overall sequence identity between the two proteins (22.6%), there are several conserved residues in the binding site that make identical interactions (three arginines and one phenylalanine) and other interactions made by similar residues in the same three-dimensional locations in the two structures. Cases such as this demonstrate that matches to even distantly related proteins can provide valid predictions about which ligands are worth considering.

## 4. Discussion

*LigSearch* is a convenient tool for identifying possible ligands for a given protein and hence can provide crystallographers with a list of candidate molecules for crystallization trials. It reduces the amount of manual searching and literature mining by providing the results automatically and conveniently clustering the resultant molecules into groups according to molecular similarity.

Arguably, the most useful matches are those that come from protein–ligand complexes in the PDB. Even if the proteins are distant relatives, the match can identify likely binding residues and indicate where the ligand might bind. The clusters represent a set of molecules that are all at least 40% similar to each other. This does not imply that they bind in the same binding site in a protein, as the clustering is performed based on molecular similarity and not three-dimensional location.

These clusters provide a very good way to identify any potential molecular frameworks that might bind to the protein. In addition, the ChEBI and ChEMBL results provide a good enrichment of the main framework in each cluster. In this example, one of the clusters has thymidine 5′-diphosphate as the highest scoring compound. From the ChEBI and ChEMBL results, another six variants such as 5′-thymidilic acid and thymidine triphosphate were added.

When looking at all of the clusters found for our example, it becomes clear that there are a large number of different molecular frameworks present. The highest scoring clusters tend to contain the substrate/product molecules as well as various versions of these molecules. A number of clusters consist of molecules commonly found in crystallization solutions, such as sulfate, phosphate and acetic acid. These molecules are difficult to exclude as they might be involved in the protein function. Some of the clusters contain highly reactive/unstable molecules such as phosphorus pentachloride and 3*H*-phosphole. Owing to the nature of the ChEBI and ChEMBL databases and their annotation, these molecules will be included in the results but will usually cluster together. This makes it easier for the crystallographer to disregard them.

The ordering of the PDB ligand hits within each cluster relies on a somewhat arbitrary scoring scheme. The rationale for the weights assigned to each interaction seems reasonable, although it would require a great deal of experimental testing to try to optimize it. Possibly it cannot, and maybe it need not, be optimized. Larger ligands tend to give a better score as they usually make more interactions with the protein, but this in itself may be suggestive of a good candidate for binding to the query protein. We welcome any collaborations willing to help with further experimental testing and ranking improvements.

Some of the searches return results for highly volatile or unstable compounds as well as compounds known to be insoluble in water. Hence, one of the improvements that is planned for the future is a more chemistry-aware filter. The implemention of a smart rule-based system, in combination with other parameters such as logP, would improve the results by removing molecules that are unlikely to be of any practical or biological use. An additional improvement, currently in the planning stages, is that all hits found should be screened against the ZINC database, checking whether the compound is purchasable.

Another potential use for *LigSearch* might be to tackle the ‘unknown ligand problem’ in which a protein structure solved by X-ray crystallography is found to have mystery density belonging to some unknown molecule in the binding site. By submitting the sequence and/or structure to the *LigSearch* server, the homology searches might provide clues to the identity of the ligand.

## Supplementary Material

A table containing extensive validation data for 200 enzymes and their substrates.. DOI: 10.1107/S0907444913022294/rr5044sup1.html

## Figures and Tables

**Table 1:** The 26 molecules manually selected in February 2012 for crystallization trials of *S. aureus* thymidylate synthase (UniProt ID P65248) and used here for testing the *LigSearch* results The molecules that were trialled are shown in bold and the two from which crystal structures were obtained are annotated with footnotes. The molecules have been grouped here by the three *LigSearch* clusters that they occurred in: *LigSearch* clusters 1, 3 and 15. The rightmost columns show details of the PDB entry from which the *LigSearch* match came.

Candidate compound	*LigSearch*	PDB code (chain)	Score	Sequence identity (%)
*LigSearch* cluster 1				
*P* ^1^-(5′-Adenosyl)-*P* ^5^-(5′-thymidyl)pentaphosphate (Fig. 4 ▶ *a*)	T5A	4tmk (*A*)	29/33	34.0
*P* ^1^-(5′-Adenosyl)-*P* ^5^-[5′-(3′-azido-3′-deoxythymidyl)] pentaphosphate	Z5A	5tmp (*A*)	25/29	34.0
Phosphoaminophosphonic acid adenylate ester	ANP	1nmy (*A*)	23/30	22.6
**Adenosine 5′-diphosphate**	ADP	2cdn (*A*)	23/32	24.3
**Adenosine 5′-triphosphate**	ATP	1e2q (*A*)	18/27	22.6
**2′-Deoxyguanosine 5′-triphosphate**	DGT	2vp2 (*A*)	18/38	26.2
**Guanosine 5′-monophosphate**	5GP	1ex7	7/–	23.7
*LigSearch* cluster 3				
**Thymidine 5′-diphosphate** (Fig. 4 ▶ *b*)	TYD	3hjn (*A*)	24/27	41.8
**Thymidine 5′-phosphate** †	TMP	2ccg (*A*)	21/21	100.0
Thymidine 5′-triphosphate	TTP	2vp0 (*A*)	21/37	26.2
2′-Deoxycytidine 5′-triphosphate	DCP	2vp4 (*A*)	20/34	26.2
**Thymidine**	THM	4esh (*A*)	13/15	35.9
3′-Deoxythymidine 5′-monophosphate	2DT	1nn0 (*A*)	11/14	22.6
**3′-Azido-3′-deoxythymidine 5′-monophosphate** ‡	ATM	1e99 (*A*)	11/19	22.6
3′-Fluoro-3′-deoxythymidine monophosphate	FDM	1nmx (*A*)	11/14	22.6
**2′-Deoxycytidine**	DCZ	1j90 (*A*)	10/22	22.7
**(*E*)-5-(2-Bromovinyl)-2′-deoxyuridine 5′-monophosphate**	BVP	2w0s (*A*)	8/12	25.3
5-Hydroxymethyluridine 2′-deoxy-5′-monophosphate	5HU	1mrs (*A*)	8/15	28.6
3′-Deoxy-3′-aminothymidine monophosphate	NYM	1nmz (*A*)	8/15	22.6
2′,3′-Dideoxycytidine 5′-monophosphate	DOC	2vp9 (*A*)	6/20	25.8
5-Bromovinyldeoxyuridine	BVD	2vqs (*A*)	5/11	26.8
**Gemcitabine**	GEO	2vpp (*A*)	5/17	26.7
**5-Fluorouridine 5′-monophosphate**	5FU	2vp6 (*A*)	5/19	25.8
*LigSearch* cluster 15				
4-(2-Hydroxyethyl)-1-piperazineethanesulfonic acid (Fig. 4 ▶ *c*)	EPE	2plr (*A*)	6/9	28.3
Not found by *LigSearch*				
**3′-Azido-3′-deoxythymidine**	—	—	—	—
Diphosphate	—	—	—	—

† Structure solved as PDB entry 4dwj.

‡ Structure solved as PDB entry 4eaq.

## Footnotes

1 Supplementary material has been deposited in the IUCr electronic archive (Reference: RR5044). Services for accessing this material are described at the back of the journal.
