# Supplementary material for: LigSearch: a knowledge-based web server to identify likely ligands for a protein target
Source: Acta Crystallogr D Biol Crystallogr. 2013 Nov 19;69(Pt 12):2395–402. doi: 10.1107/S0907444913022294 (PMC3852652; doi:10.1107/S0907444913022294)
Supplement: Supplementary file 1 [file d-69-02395-sup1.html]

LigSearch:�Supplementary�information


# LigSearch:�Supplementary�information

|  |
| --- |
| Results of the LigSearch validation run against a randomly selected set of 207 enzymes that do not have a 3D structure in the PDB (as of July 2013). Each is represented by a randomly chosen UniProt sequence. The KEGG molecules are the molecules known to bind to the enzyme, being its reactants, products or cofactors. The closest match to each of these molecules, returned by LigSearch from the structures in the PDB, is shown together with the LigSearch cluster it appeared in, and its rank within that cluster. The molecular similarity between the known binding molecule and the PDB ligand is given next, as computed by the SMSD program. The PDB code (and chain) follow, with its similarity to the search sequence quantified by its percentage sequence identity and E-value. The final three columns show the LigSearch scores. The first is the score computed from the number and type of protein-ligand interactions in the PDB structure, and adjusted to reflect the similarity of the interacting residues to the corresponding residues in the query sequence. The second score, the Max score, shows the maximum score that would be obtained if the corresponding residues were of identical amino acid types. The final column gives the ligand score as a percentage of the maximum score. |

|  |  |  |  |  |  |  |  |  |  |  |  |  |
| --- | --- | --- | --- | --- | --- | --- | --- | --- | --- | --- | --- | --- |
| E.C. Number | UniProt id | KEGG molecule (enzyme reactant, product and/or cofactor) | Closest PDB ligand matched | Cluster | Rank in cluster | **Molec simil** | PDB code | Seq id(%) | E-value | Score, S | Max score | %-tage |
| 6.2.1.14 | A6VHG3 | AMP | COA | 1 | 1 | 0.810 | 2o28(A) | 24.3 | 0.52 | 24 | 45 | 53.333 |
|  |  | ATP | COA | 1 | 1 | 0.839 | 2o28(A) | 24.3 | 0.52 | 24 | 45 | 53.333 |
|  |  | coenzyme A | COA | 1 | 1 | 1.000 | 2o28(A) | 24.3 | 0.52 | 24 | 45 | 53.333 |
|  |  | pimeloyl-CoA | COA | 1 | 1 | 0.944 | 2o28(A) | 24.3 | 0.52 | 24 | 45 | 53.333 |
|  |  | pimelic acid | GLP | 7 | 2 | 0.080 | 3cxq(A) | 24.3 | 0.52 | 7 | 17 | 41.176 |
|  |  | diphosphoric acid | GLP | 7 | 2 | 0.043 | 3cxq(A) | 24.3 | 0.52 | 7 | 17 | 41.176 |
|  |  | diphosphate(4-) | GLP | 7 | 2 | 0.043 | 3cxq(A) | 24.3 | 0.52 | 7 | 17 | 41.176 |
| 2.7.1.166 | B2I5R8 | ATP | ADP | 2 | 1 | 0.981 | 4gyi(A) | 30.9 | 1.3 | 4 | 10 | 40 |
|  |  | ADP | ADP | 2 | 1 | 1 | 4gyi(A) | 30.9 | 1.3 | 4 | 10 | 40 |
| 2.1.1.189 | B2K9X0 | S-adenosyl-L-methionine | SAM | 1 | 2 | 1 | 4dcm(A) | 26.7 | 48 | 24 | 35 | 68.571 |
|  |  | S-adenosyl-L-homocysteine | SAH | 1 | 1 | 1 | 1l3i(A) | 25.2 | 2.1 | 27 | 52 | 51.923 |
| 3.1.4.14 | B5R6Q5 | D-pantetheine 4'-phosphate | SAH | 1 | 1 | 0.125 | 3ou2(A) | 23.8 | 4.8 | 7 | 43 | 16.279 |
|  |  | pantetheine 4'-phosphate | SAH | 1 | 1 | 0.125 | 3ou2(A) | 23.8 | 4.8 | 7 | 43 | 16.279 |
| 1.1.1.326 | F1SWA0 | NADH | NAI | 1 | 5 | 1.000 | 3vdr(A) | 30.1 | 7.50E-16 | 49 | 65 | 75.385 |
|  |  | NAD(+) | NAD | 1 | 2 | 0.998 | 3ijr(A) | 29.4 | 2.10E-10 | 52 | 81 | 64.198 |
|  |  | zerumbone | AOI | 47 | 1 | 0.381 | 1yb1(A) | 26 | 2.00E-08 | 5 | 6 | 83.333 |
| 1.4.3.20 | F2JXJ3 | L-allysine | ACT | 1 | 1 | 0.235 | 3l2n(A) | 27.2 | 31 | 7 | 7 | 100 |
|  |  | L-lysine | ACT | 1 | 1 | 0.242 | 3l2n(A) | 27.2 | 31 | 7 | 7 | 100 |
| 2.3.1.140 | G0LD36 | coenzyme A | COA | 1 | 2 | 1 | 2zba(A) | 25.4 | 2.00E-05 | 20 | 40 | 50 |
|  |  | caffeoyl-CoA | MLC | 1 | 1 | 0.925 | 2e1t(A) | 27 | 0.93 | 25 | 49 | 51.02 |
|  |  | (R)-rosmarinic acid | B2S | 6 | 2 | 0.221 | 3b2s(A) | 22.7 | 0.00019 | -1 | 22 | -4.545 |
|  |  | (S)-rosmarinic acid | B2S | 6 | 2 | 0.221 | 3b2s(A) | 22.7 | 0.00019 | -1 | 22 | -4.545 |
| 1.3.1.96 | G0Y287 | squalene | DH7 | 13 | 1 | 0.558 | 3nri(A) | 22.1 | 0.015 | 21 | 32 | 65.625 |
|  |  | presqualene diphosphate | PS7 | 1 | 1 | 1.000 | 3npr(A) | 23.5 | 0.016 | 44 | 57 | 77.193 |
|  |  | diphosphoric acid | POP | 19 | 1 | 0.923 | 4ea0(A) | 23.5 | 0.016 | 18 | 19 | 94.737 |
|  |  | diphosphate(4-) | POP | 19 | 1 | 1.000 | 4ea0(A) | 23.5 | 0.016 | 18 | 19 | 94.737 |
|  |  | NADPH | D3A | 7 | 1 | 0.434 | 3v66(A) | 46.6 | 9.00E-59 | 28 | 35 | 80 |
|  |  | NADP(+) | D3A | 7 | 1 | 0.428 | 3v66(A) | 46.6 | 9.00E-59 | 28 | 35 | 80 |
| 4.2.1.54 | G3KIM3 | acryloyl-CoA | NAD | 2 | 1 | 0.767 | 3uwr(A) | 28.4 | 23 | 12 | 30 | 40 |
| 1.14.11.20 | O04847 | 2-oxoglutaric acid | AKG | 6 | 1 | 1.000 | 2brt(A) | 29.9 | 5.60E-30 | 22 | 25 | 88 |
|  |  | succinic acid | SIN | 6 | 2 | 1.000 | 1gp6(A) | 29.9 | 5.60E-30 | 15 | 17 | 88.235 |
|  |  | 17-O-deacetylvindoline | APV | 7 | 1 | 0.318 | 1odn(A) | 24.2 | 6.5 | 19 | 28 | 67.857 |
|  |  | deacetoxyvindoline | A14 | 7 | 3 | 0.320 | 2jb4(A) | 24.2 | 6.7 | 15 | 31 | 48.387 |
| 1.2.1.67 | O05619 | NADH | NAI | 1 | 5 | 1 | 2j6l(A) | 26.1 | 1.30E-10 | 37 | 59 | 62.712 |
|  |  | NAD(+) | NAD | 1 | 2 | 0.998 | 1a4z(A) | 35.3 | 8.20E-22 | 50 | 64 | 78.125 |
|  |  | vanillin | I1E | 8 | 2 | 0.408 | 3szb(A) | 30 | 1.50E-12 | 8 | 18 | 44.444 |
|  |  | vanillic acid | I1E | 8 | 2 | 0.408 | 3szb(A) | 30 | 1.50E-12 | 8 | 18 | 44.444 |
| 5.4.99.16 | O06458 | alpha,alpha-trehalose | TRE | 1 | 36 | 1 | 2bhy(A) | 35.2 | 1.50E-06 | 1 | 10 | 10 |
|  |  | maltose | GLC-GLC | 1 | 7 | 1 | 1pj9(A) | 29.5 | 2.1 | 22 | 37 | 59.459 |
| 1.1.1.65 | O14295 | NADPH | NDP | 1 | 1 | 1 | 1qrq(A) | 20.2 | 3.50E-09 | 52 | 81 | 64.198 |
|  |  | NADP(+) | NAP | 1 | 2 | 0.998 | 3h7u(A) | 22.9 | 2.5 | 49 | 72 | 68.056 |
|  |  | pyridoxal | PDN | 7 | 1 | 0.159 | 3eau(A) | 20.2 | 3.50E-09 | 7 | 17 | 41.176 |
|  |  | pyridoxine | PDN | 7 | 1 | 0.152 | 3eau(A) | 20.2 | 3.50E-09 | 7 | 17 | 41.176 |
| 2.7.7.30 | O14772 | GTP | AMP | 1 | 1 | 0.669 | 2f17(A) | 25.4 | 18 | 23 | 35 | 65.714 |
|  |  | GDP-L-fucose | AMP | 1 | 1 | 0.623 | 2f17(A) | 25.4 | 18 | 23 | 35 | 65.714 |
|  |  | diphosphoric acid | PO4 | 5 | 1 | 0.461 | 1kv8(A) | 26.7 | 12 | 6 | 15 | 40 |
|  |  | diphosphate(4-) | PO4 | 5 | 1 | 0.538 | 1kv8(A) | 26.7 | 12 | 6 | 15 | 40 |
|  |  | L-fucopyranose 1-phosphate | LG6 | 6 | 6 | 0.294 | 1q6o(A) | 26.7 | 12 | 3 | 37 | 8.108 |
| 4.2.3.16 | O22340 | diphosphoric acid | POP | 3 | 1 | 0.923 | 1n23(A) | 32.4 | 1.30E-29 | 21 | 30 | 70 |
|  |  | diphosphate(4-) | POP | 3 | 1 | 1.000 | 1n23(A) | 32.4 | 1.30E-29 | 21 | 30 | 70 |
|  |  | geranyl diphosphate | FGG | 2 | 3 | 0.743 | 3p5r(A) | 39.9 | 7.80E-55 | 18 | 39 | 46.154 |
| 6.2.1.12 | O24146 | AMP | AMP | 1 | 3 | 1 | 3a9v(A) | 80 | 2.90E-33 | 35 | 37 | 94.595 |
|  |  | ATP | ATP | 1 | 4 | 1 | 4gxq(A) | 34.6 | 3.20E-07 | 34 | 49 | 69.388 |
|  |  | coenzyme A | COA | 1 | 20 | 1 | 3gpc(A) | 32.4 | 1.30E-07 | 18 | 41 | 43.902 |
|  |  | 4-coumaroyl-CoA | BCO | 1 | 12 | 0.935 | 3eq6(A) | 22.5 | 0.017 | 23 | 48 | 47.917 |
|  |  | diphosphoric acid | POP | 25 | 1 | 0.923 | 3lnv(A) | 26.9 | 3.90E-05 | 4 | 10 | 40 |
|  |  | diphosphate(4-) | POP | 25 | 1 | 1 | 3lnv(A) | 26.9 | 3.90E-05 | 4 | 10 | 40 |
|  |  | trans-4-coumaric acid | PHE | 9 | 1 | 0.407 | 1amu(A) | 31.1 | 0.0026 | 10 | 24 | 41.667 |
| 2.5.1.84 | O24743 | geranyl diphosphate | GPP | 1 | 4 | 1 | 2e8x(B) | 25 | 2.30E-10 | 20 | 31 | 64.516 |
|  |  | all-trans-nonaprenyl diphosphate | GRG | 1 | 1 | 1 | 2q80(A) | 25.6 | 2.80E-11 | 37 | 46 | 80.435 |
|  |  | isopentenyl diphosphate | IPE | 15 | 1 | 1 | 1rqj(A) | 33.6 | 7.30E-08 | 25 | 26 | 96.154 |
|  |  | diphosphoric acid | PPV | 23 | 1 | 1 | 3krf(A) | 32.2 | 0.002 | 16 | 16 | 100 |
|  |  | diphosphate(4-) | POP | 23 | 2 | 1 | 4h5d(F) | 24.2 | 0.0038 | 15 | 16 | 93.75 |
| 2.3.1.182 | O26819 | D-citramalic acid | OAA | 1 | 6 | 0.875 | 1rqe(A) | 20.9 | 0.71 | 9 | 20 | 45 |
|  |  | coenzyme A | ACO | 2 | 3 | 0.979 | 3bli(A) | 34.7 | 2.40E-22 | 14 | 22 | 63.636 |
|  |  | acetyl-CoA | ACO | 2 | 3 | 1 | 3bli(A) | 34.7 | 2.40E-22 | 14 | 22 | 63.636 |
|  |  | pyruvic acid | PYR | 4 | 1 | 1 | 3blf(A) | 34.7 | 5.80E-21 | 15 | 19 | 78.947 |
| 1.97.1.10 | O42411 | 3,3',5-triiodo-L-thyronine | 2PG | 1 | 1 | 0.094 | 3uje(A) | 25.4 | 1.2 | 12 | 47 | 25.532 |
|  |  | L-thyroxine | 2PG | 1 | 1 | 0.094 | 3uje(A) | 25.4 | 1.2 | 12 | 47 | 25.532 |
| 3.5.3.9 | O49434 | allantoic acid | 1AL | 7 | 1 | 0.977 | 1z2l(B) | 39.7 | 0.0015 | 9 | 9 | 100 |
|  |  | 2-ureidoglycine | 1AL | 7 | 1 | 0.773 | 1z2l(B) | 39.7 | 0.0015 | 9 | 9 | 100 |
| 1.3.1.29 | O52384 | NADH | NAI | 1 | 5 | 1 | 3vdr(A) | 28.6 | 2.10E-14 | 48 | 65 | 73.846 |
|  |  | NAD(+) | NAD | 1 | 3 | 1 | 3ijr(A) | 29.8 | 1.20E-11 | 56 | 81 | 69.136 |
|  |  | naphthalene-1,2-diol | CYH | 50 | 1 | 0.438 | 1b2l(A) | 27.2 | 0.32 | 4 | 10 | 40 |
|  |  | (1R,2S)-1,2-dihydronaphthalene-1,2-diol | C0R | 5 | 1 | 0.364 | 1y5r(A) | 28.7 | 9.90E-07 | 15 | 24 | 62.5 |
| 1.21.4.4 | O69407 | acetyl dihydrogen phosphate | 1GP | 18 | 1 | 0.421 | 2f6x(A) | 26.7 | 0.91 | 1 | 3 | 33.333 |
|  |  | glycine betaine | CIT | 19 | 1 | 0.231 | 2f6u(A) | 26.7 | 0.91 | 1 | 3 | 33.333 |
|  |  | trimethylamine | GOL | 12 | 2 | 0.083 | 4iqf(A) | 22 | 5.4 | 1 | 11 | 9.091 |
|  |  | phosphoric acid | 1GP | 18 | 1 | 0.207 | 2f6x(A) | 26.7 | 0.91 | 1 | 3 | 33.333 |
| 3.1.6.6 | O69787 | sulfuric acid | SO4 | 2 | 1 | 0.875 | 3ed4(A) | 28.3 | 1.60E-14 | 13 | 17 | 76.471 |
|  |  | choline | EDO | 18 | 1 | 0.357 | 3b5q(A) | 26.1 | 8.30E-06 | 7 | 12 | 58.333 |
|  |  | choline sulfate | SO4 | 2 | 1 | 0.258 | 3ed4(A) | 28.3 | 1.60E-14 | 13 | 17 | 76.471 |
| 1.14.99.33 | O81931 | linoleic acid | FLC | 60 | 1 | 0.286 | 1c5r(A) | 21.7 | 32 | 1 | 3 | 33.333 |
|  |  | crepenynic acid | FLC | 60 | 1 | 0.222 | 1c5r(A) | 21.7 | 32 | 1 | 3 | 33.333 |
|  |  | crepenynate | FLC | 60 | 1 | 0.222 | 1c5r(A) | 21.7 | 32 | 1 | 3 | 33.333 |
| 1.4.99.5 | O85228 | glycine | SAR | 6 | 3 | 0.778 | 3qse(A) | 33.3 | 0.083 | 1 | 3 | 33.333 |
| 2.2.1.3 | O93884 | D-xylulose 5-phosphate | R5P | 3 | 1 | 0.852 | 2r5n(A) | 38.4 | 2.10E-21 | 20 | 20 | 100 |
|  |  | glycerone | GOL | 5 | 2 | 0.75 | 3m34(A) | 37.7 | 6.30E-47 | 8 | 18 | 44.444 |
|  |  | D-glyceraldehyde 3-phosphate | E4P | 3 | 2 | 0.66 | 1ngs(B) | 40.6 | 5.30E-35 | 12 | 12 | 100 |
| 2.7.1.119 | P09979 | hygromycin B | SRY | 2 | 4 | 0.547 | 3hav(A) | 21.6 | 27 | 7 | 19 | 36.842 |
|  |  | ATP | ATP | 1 | 7 | 1 | 3hav(A) | 21.6 | 18 | 4 | 7 | 57.143 |
|  |  | ADP | ADP | 1 | 2 | 1 | 2bkk(A) | 28.4 | 30 | 11 | 18 | 61.111 |
| 2.7.7.46 | P0AE04 | diphosphoric acid | CTP | 1 | 2 | 0.025 | 3h3a(A) | 27.1 | 23 | 3 | 11 | 27.273 |
|  |  | diphosphate(4-) | \_CL | 8 | 1 | 0.071 | 4e8j(A) | 24 | 0.011 | 1 | 3 | 33.333 |
| 1.3.1.58 | P0C622 | NADH | NAI | 1 | 3 | 1 | 2dkn(A) | 29.8 | 1.40E-06 | 45 | 65 | 69.231 |
|  |  | NAD(+) | NAD | 1 | 2 | 0.998 | 3lqf(A) | 37.4 | 5.40E-25 | 45 | 63 | 71.429 |
|  |  | 2,3-dihydroxy-p-cumic acid | SS2 | 54 | 1 | 0.557 | 1zk0(A) | 33.6 | 1.60E-17 | 2 | 8 | 25 |
|  |  | 2,3-dihydroxy-p-cumate | SS2 | 54 | 1 | 0.548 | 1zk0(A) | 33.6 | 1.60E-17 | 2 | 8 | 25 |
|  |  | cis-5,6-dihydroxy-4-isopropylcyclohexa-1,3-dienecarboxylic acid | TES | 39 | 2 | 0.397 | 1jtv(A) | 25.9 | 7.70E-06 | 1 | 15 | 6.667 |
| 1.14.13.59 | P11295 | NADPH | NDP | 1 | 6 | 1.000 | 2x99(A) | 28.1 | 15 | 6 | 43 | 13.953 |
|  |  | NADP(+) | NAP | 1 | 5 | 0.998 | 4b63(A) | 38.1 | 4.00E-05 | 6 | 46 | 13.043 |
|  |  | L-lysine | LYS | 7 | 3 | 0.970 | 4b64(A) | 35.7 | 4.80E-05 | 5 | 18 | 27.778 |
|  |  | N(6)-hydroxy-L-lysine | LYS | 7 | 3 | 0.750 | 4b64(A) | 35.7 | 4.80E-05 | 5 | 18 | 27.778 |
| 3.1.1.59 | P12992 | (2E,6E,10R,11S)-10,11-epoxy-3,7,11-trimethyltrideca-2,6-dienoic acid | CLL | 18 | 1 | 0.319 | 1cle(A) | 29.5 | 1.90E-15 | 17 | 48 | 35.417 |
| 2.4.1.52 | P13484 | UDP | UDP | 1 | 2 | 1 | 2xmp(A) | 24.6 | 0.33 | 21 | 29 | 72.414 |
| 2.7.8.12 | P13485 | CMP | FMN | 2 | 1 | 0.362 | 2d5m(A) | 27.1 | 27 | 22 | 58 | 37.931 |
| 1.1.1.342 | P14168 | NADPH | NDP | 1 | 2 | 1.000 | 3i6q(A) | 25.8 | 0.54 | 28 | 53 | 52.83 |
|  |  | NADP(+) | NAP | 1 | 1 | 0.998 | 3i52(A) | 25.8 | 0.55 | 33 | 64 | 51.562 |
|  |  | CDP-3,6-dideoxy-alpha-D-glucose | UPG | 1 | 12 | 0.782 | 1i3k(A) | 21.8 | 39 | -1 | 42 | -2.381 |
|  |  | CDP-4-dehydro-3,6-dideoxy-alpha-D-glucose | UPG | 1 | 12 | 0.774 | 1i3k(A) | 21.8 | 39 | -1 | 42 | -2.381 |
| 6.2.1.19 | P14286 | AMP | H37 | 2 | 1 | 0.17 | 1cvr(A) | 27 | 22 | 8 | 30 | 26.667 |
|  |  | ATP | H37 | 2 | 1 | 0.174 | 1cvr(A) | 27 | 22 | 8 | 30 | 26.667 |
|  |  | diphosphoric acid | H37 | 2 | 1 | 0.015 | 1cvr(A) | 27 | 22 | 8 | 30 | 26.667 |
|  |  | diphosphate(4-) | H37 | 2 | 1 | 0.015 | 1cvr(A) | 27 | 22 | 8 | 30 | 26.667 |
| 2.4.2.37 | P14299 | NAD(+) | HEC | 4 | 2 | 0.468 | 3ej8(A) | 39.6 | 1.8 | -11 | 32 | -34.375 |
|  |  | nicotinamide | AT6 | 12 | 1 | 0.243 | 3e68(A) | 32.1 | 16 | -9 | 22 | -40.909 |
| 1.1.99.10 | P18172 | D-glucono-1,5-lactone | MAN | 8 | 1 | 0.845 | 1kdg(A) | 27.3 | 0.004 | 5 | 7 | 71.429 |
|  |  | D-glucopyranose | MAN | 8 | 1 | 1 | 1kdg(A) | 27.3 | 0.004 | 5 | 7 | 71.429 |
| 2.7.1.103 | P18623 | ATP | ATP | 3 | 1 | 1 | 4ej7(A) | 33.3 | 2.7 | 13 | 19 | 68.421 |
|  |  | ADP | ADP | 3 | 3 | 1 | 4dca(A) | 23.6 | 0.25 | 6 | 8 | 75 |
|  |  | viomycin | GDP | 3 | 2 | 0.401 | 3tdv(A) | 24.3 | 1.10E-05 | 9 | 14 | 64.286 |
|  |  | O-phosphoviomycin | GDP | 3 | 2 | 0.424 | 3tdv(A) | 24.3 | 1.10E-05 | 9 | 14 | 64.286 |
| 4.5.1.3 | P21161 | dichloromethane | \_CL | 22 | 1 | 0.2 | 3ubl(A) | 32.1 | 6.3 | 3 | 3 | 100 |
| 2.1.1.131 | P21640 | S-adenosyl-L-methionine | SAH | 1 | 1 | 1 | 2zvb(A) | 38.2 | 4.40E-11 | 37 | 47 | 78.723 |
|  |  | S-adenosyl-L-homocysteine | SAH | 1 | 1 | 1 | 2zvb(A) | 38.2 | 4.40E-11 | 37 | 47 | 78.723 |
|  |  | precorrin-3B | UP2 | 9 | 1 | 0.417 | 2ybq(A) | 28.4 | 0.15 | 3 | 13 | 23.077 |
|  |  | precorrin-4 | UP2 | 9 | 1 | 0.402 | 2ybq(A) | 28.4 | 0.15 | 3 | 13 | 23.077 |
| 2.3.1.165 | P22367 | NADPH | NDP | 1 | 1 | 1 | 3slk(A) | 33.1 | 1.00E-10 | 49 | 60 | 81.667 |
|  |  | coenzyme A | COZ | 1 | 5 | 1 | 2g2z(A) | 30.6 | 2.6 | 15 | 27 | 55.556 |
|  |  | acetyl-CoA | COZ | 1 | 5 | 0.979 | 2g2z(A) | 30.6 | 2.6 | 15 | 27 | 55.556 |
|  |  | NADP(+) | NAP | 1 | 2 | 0.998 | 1edo(A) | 22.1 | 0.31 | 35 | 74 | 47.297 |
|  |  | malonyl-CoA | COZ | 1 | 5 | 0.96 | 2g2z(A) | 30.6 | 2.6 | 15 | 27 | 55.556 |
|  |  | 6-methylsalicylic acid | N32 | 3 | 3 | 0.185 | 4f32(A) | 25 | 0.0061 | 25 | 41 | 60.976 |
|  |  | 6-methylsalicylate | ACT | 7 | 2 | 0.191 | 2hg4(A) | 35.5 | 4.30E-11 | 12 | 19 | 63.158 |
| 1.14.11.11 | P24397 | 2-oxoglutaric acid | AKG | 4 | 1 | 1 | 2brt(A) | 27.1 | 6.60E-29 | 24 | 25 | 96 |
|  |  | succinic acid | SIN | 4 | 3 | 1 | 1gp6(A) | 27.1 | 6.60E-29 | 15 | 17 | 88.235 |
|  |  | (S)-atropine | W2X | 2 | 4 | 0.25 | 1w3x(A) | 23.4 | 0.012 | 23 | 42 | 54.762 |
|  |  | (6S)-6-hydroxyhyoscyamine | PN1 | 6 | 3 | 0.261 | 1w2n(A) | 22.3 | 1.6 | 9 | 18 | 50 |
| 6.3.2.26 | P25464 | AMP | AMP | 1 | 4 | 1 | 1amu(A) | 38.5 | 3.00E-08 | 35 | 40 | 87.5 |
|  |  | ATP | ATP | 1 | 1 | 1 | 3lgx(A) | 26.9 | 0.0046 | 43 | 45 | 95.556 |
|  |  | L-valine | LEU | 14 | 1 | 0.84 | 2vsq(A) | 31.1 | 7.80E-08 | 10 | 18 | 55.556 |
|  |  | L-2-aminoadipic acid | LEU | 14 | 1 | 0.735 | 2vsq(A) | 31.1 | 7.80E-08 | 10 | 18 | 55.556 |
|  |  | diphosphoric acid | POP | 21 | 1 | 0.923 | 3lnv(A) | 25.3 | 0.029 | 8 | 12 | 66.667 |
|  |  | diphosphate(4-) | POP | 21 | 1 | 1 | 3lnv(A) | 25.3 | 0.029 | 8 | 12 | 66.667 |
|  |  | L-cysteine | LEU | 14 | 1 | 0.531 | 2vsq(A) | 31.1 | 7.80E-08 | 10 | 18 | 55.556 |
|  |  | N-[(5S)-5-amino-5-carboxypentanoyl]-L-cysteinyl-D-valine | ALA-MET-SEP-PHE-GLN-SER | 51 | 1 | 0.358 | 4dau(B) | 28.7 | 34 | 2 | 17 | 11.765 |
| 2.7.8.6 | P26406 | UMP | HEM-ACT | 2 | 1 | 0.338 | 2bmm(A) | 32.8 | 17 | 19 | 48 | 39.583 |
|  |  | UDP-D-galactose | HEM-ACT | 2 | 1 | 0.395 | 2bmm(A) | 32.8 | 17 | 19 | 48 | 39.583 |
|  |  | ditrans,polycis-undecaprenyl phosphate | MRD | 23 | 1 | 0.147 | 3cu2(A) | 28.4 | 24 | 1 | 6 | 16.667 |
|  |  | alpha-D-galactosyl undecaprenyl diphosphate | NAG | 18 | 1 | 0.332 | 1npm(A) | 27.7 | 29 | 2 | 4 | 50 |
| 2.3.1.170 | P26690 | NADPH | NDP | 1 | 2 | 1 | 2vdg(A) | 41.3 | 1.70E-46 | 56 | 78 | 71.795 |
|  |  | coenzyme A | NA7 | 1 | 4 | 0.846 | 2wzm(A) | 31 | 3.60E-13 | 29 | 47 | 61.702 |
|  |  | 4-coumaroyl-CoA | NA7 | 1 | 4 | 0.776 | 2wzm(A) | 31 | 3.60E-13 | 29 | 47 | 61.702 |
|  |  | NADP(+) | NAP | 1 | 1 | 0.998 | 1zgd(A) | 85.1 | 1.50E-123 | 64 | 65 | 98.462 |
|  |  | malonyl-CoA | NA7 | 1 | 4 | 0.817 | 2wzm(A) | 31 | 3.60E-13 | 29 | 47 | 61.702 |
|  |  | isoliquiritigenin | IU5 | 50 | 3 | 0.337 | 1ihi(A) | 37 | 1.00E-32 | 5 | 21 | 23.81 |
| 2.4.1.56 | P27242 | UDP | ADP | 1 | 4 | 0.565 | 4a7o(A) | 34.8 | 0.45 | 7 | 27 | 25.926 |
|  |  | UDP-N-acetyl-alpha-D-glucosamine | AFH | 1 | 1 | 0.62 | 2h1h(A) | 34.1 | 4.2 | 11 | 41 | 26.829 |
| 1.1.1.244 | P31005 | NADH | NAD | 1 | 1 | 0.876 | 2bl4(A) | 40.7 | 6.40E-23 | 54 | 67 | 80.597 |
|  |  | NAD(+) | NAD | 1 | 1 | 0.998 | 2bl4(A) | 40.7 | 6.40E-23 | 54 | 67 | 80.597 |
| 2.1.1.100 | P32584 | S-adenosyl-L-methionine | SAH | 1 | 1 | 0.998 | 4a2n(B) | 37.6 | 3.20E-09 | 30 | 44 | 68.182 |
|  |  | S-adenosyl-L-homocysteine | SAH | 1 | 1 | 1 | 4a2n(B) | 37.6 | 3.20E-09 | 30 | 44 | 68.182 |
| 2.7.1.150 | P34756 | ATP | ATP | 2 | 1 | 1 | 1kvk(A) | 22.2 | 9.6 | 17 | 44 | 38.636 |
|  |  | ADP | ATP | 2 | 1 | 0.981 | 1kvk(A) | 22.2 | 9.6 | 17 | 44 | 38.636 |
| 1.1.1.61 | P38945 | NADH | NAD | 1 | 1 | 0.876 | 2bl4(A) | 27.3 | 0.6 | 49 | 67 | 73.134 |
|  |  | NAD(+) | NAD | 1 | 1 | 0.998 | 2bl4(A) | 27.3 | 0.6 | 49 | 67 | 73.134 |
|  |  | 4-hydroxybutyric acid | PGO | 2 | 1 | 0.412 | 1rrm(A) | 27.3 | 0.6 | 13 | 13 | 100 |
|  |  | succinic semialdehyde | PGO | 2 | 1 | 0.389 | 1rrm(A) | 27.3 | 0.6 | 13 | 13 | 100 |
| 1.1.1.57 | P39160 | NADH | NAI | 1 | 1 | 1 | 4im7(A) | 56.7 | 1.00E-67 | 40 | 46 | 86.957 |
|  |  | NAD(+) | NAD | 1 | 2 | 1 | 1lj8(A) | 43.4 | 3.00E-52 | 35 | 46 | 76.087 |
|  |  | D-mannonic acid | CS2 | 2 | 1 | 1 | 4im7(A) | 56.7 | 5.20E-68 | 30 | 30 | 100 |
|  |  | D-mannonate | CS2 | 2 | 1 | 0.968 | 4im7(A) | 56.7 | 5.20E-68 | 30 | 30 | 100 |
|  |  | D-fructofuranuronic acid | CS2 | 2 | 1 | 0.278 | 4im7(A) | 56.7 | 5.20E-68 | 30 | 30 | 100 |
| 3.2.1.93 | P39795 | alpha,alpha-trehalose 6-phosphate | TRE | 1 | 25 | 0.775 | 2bhy(A) | 35.4 | 7.90E-06 | 2 | 10 | 20 |
|  |  | D-glucopyranose | BGC | 2 | 2 | 1 | 2pwf(A) | 57.4 | 4.30E-30 | 31 | 35 | 88.571 |
|  |  | D-glucopyranose 6-phosphate | BGC | 2 | 2 | 0.623 | 2pwf(A) | 57.4 | 4.30E-30 | 31 | 35 | 88.571 |
| 2.5.1.90 | P44916 | 2-trans,6-trans-farnesyl diphosphate | GRG | 1 | 1 | 1 | 2q80(A) | 25.3 | 0.00028 | 38 | 46 | 82.609 |
|  |  | all-trans-octaprenyl diphosphate | GRG | 1 | 1 | 1 | 2q80(A) | 25.3 | 0.00028 | 38 | 46 | 82.609 |
|  |  | isopentenyl diphosphate | IPE | 11 | 1 | 1 | 1rqj(A) | 29.2 | 4.10E-06 | 26 | 26 | 100 |
|  |  | diphosphoric acid | PPV | 16 | 1 | 1 | 3krf(A) | 31.7 | 3.60E-11 | 16 | 16 | 100 |
|  |  | diphosphate(4-) | POP | 16 | 2 | 1 | 3oyr(A) | 39.4 | 1.50E-19 | 10 | 11 | 90.909 |
| 1.97.1.11 | P49898 | L-thyroxine | FAD | 1 | 8 | 0.112 | 2qae(A) | 30.1 | 2 | -18 | 76 | -23.684 |
|  |  | 3,3',5'-triiodothyronine | FAD | 1 | 8 | 0.112 | 2qae(A) | 30.1 | 2 | -18 | 76 | -23.684 |
| 3.6.3.34 | P49938 | ATP | ATP | 1 | 4 | 1 | 2r6g(A) | 28.6 | 1.80E-17 | 27 | 33 | 81.818 |
|  |  | ADP | ADP-BEF | 1 | 2 | 1 | 3pux(A) | 29 | 9.30E-18 | 36 | 41 | 87.805 |
|  |  | phosphoric acid | PO4 | 2 | 2 | 0.857 | 2qi9(C) | 34.1 | 1.80E-16 | 16 | 18 | 88.889 |
| 1.1.1.250 | P50167 | NADH | NAI | 1 | 5 | 1 | 3vdr(A) | 26.5 | 7.70E-10 | 45 | 65 | 69.231 |
|  |  | NAD(+) | NAD | 1 | 3 | 0.998 | 3ijr(A) | 26.9 | 2.70E-08 | 49 | 81 | 60.494 |
|  |  | D-arabinitol | SOR | 3 | 4 | 0.833 | 1uzo(A) | 26.3 | 0.021 | 10 | 20 | 50 |
|  |  | D-ribulose | SOL | 5 | 1 | 0.815 | 3ai3(A) | 29.5 | 1.10E-05 | 14 | 23 | 60.87 |
| 4.1.2.38 | P51853 | benzoin | RMN | 10 | 2 | 0.729 | 1mcz(B) | 25.3 | 3.00E-05 | 6 | 10 | 60 |
|  |  | benzaldehyde | RMN | 10 | 2 | 0.46 | 1mcz(B) | 25.3 | 3.00E-05 | 6 | 10 | 60 |
| 2.8.2.25 | P52836 | adenosine 3',5'-bismonophosphate | A3P | 1 | 3 | 1 | 2d06(A) | 29.6 | 1.50E-19 | 45 | 51 | 88.235 |
|  |  | 3'-phospho-5'-adenylyl sulfate | PPS | 1 | 1 | 1 | 2zyt(X) | 30.5 | 1.30E-20 | 51 | 59 | 86.441 |
|  |  | quercetin | 3QV | 17 | 1 | 0.351 | 3u3o(A) | 29.6 | 1.50E-19 | 8 | 20 | 40 |
|  |  | quercetin 3-sulfate | GHP-3MY-3FG-GHP-GHP-OMY-3FG | 2 | 1 | 0.363 | 3mg9(B) | 24.7 | 0.00024 | 33 | 55 | 60 |
| 2.4.2.40 | P56725 | UDP | UDP | 1 | 3 | 1 | 2vch(A) | 39.9 | 5.30E-31 | 33 | 37 | 89.189 |
|  |  | UDP-alpha-D-xylose | UPG | 1 | 2 | 0.869 | 2acw(A) | 23 | 35 | 36 | 53 | 67.925 |
|  |  | trans-zeatin | NAP | 1 | 5 | 0.196 | 2gq2(C) | 26 | 3.4 | 5 | 15 | 33.333 |
|  |  | O-beta-D-xylosylzeatin | ERY | 12 | 1 | 0.344 | 2iyf(A) | 27.7 | 1.1 | 2 | 2 | 100 |
| 3.6.3.38 | P57013 | ATP | ATP | 1 | 4 | 1 | 2fgk(A) | 23.7 | 0.024 | 28 | 34 | 82.353 |
|  |  | ADP | ADP | 1 | 3 | 1 | 2pmk(A) | 24.2 | 0.024 | 30 | 37 | 81.081 |
|  |  | phosphoric acid | PO4 | 2 | 2 | 0.857 | 2onk(A) | 25.4 | 2.9 | 14 | 18 | 77.778 |
| 2.1.1.168 | P78860 | S-adenosyl-L-methionine | SAM | 1 | 1 | 1 | 1ej0(A) | 33.5 | 9.10E-07 | 34 | 45 | 75.556 |
|  |  | S-adenosyl-L-homocysteine | SAH | 1 | 2 | 1 | 2fyt(A) | 29.1 | 24 | 14 | 44 | 31.818 |
| 1.2.7.3 | P80906 | 2-oxoglutaric acid | SO4 | 1 | 1 | 0.03 | 3on3(B) | 45.9 | 1.50E-16 | 14 | 15 | 93.333 |
|  |  | coenzyme A | SO4 | 1 | 1 | 0.011 | 3on3(B) | 45.9 | 1.50E-16 | 14 | 15 | 93.333 |
|  |  | succinyl-CoA | SO4 | 1 | 1 | 0.01 | 3on3(B) | 45.9 | 1.50E-16 | 14 | 15 | 93.333 |
| 6.2.1.33 | P86832 | ATP | ATP | 1 | 4 | 1 | 4gxq(A) | 31.2 | 0.1 | 28 | 49 | 57.143 |
|  |  | coenzyme A | COA | 1 | 21 | 1 | 1pg3(A) | 25.9 | 1.9 | 8 | 46 | 17.391 |
|  |  | 4-chlorobenzoyl-CoA | BCO | 1 | 17 | 0.941 | 3eq6(A) | 39 | 4.10E-10 | 11 | 25 | 44 |
| 3.5.99.3 | P95442 | 4-ethylamino-6-isopropylamino-1,3,5-triazin-2-ol | AOO | 3 | 1 | 0.831 | 3lsc(A) | 30.3 | 4.9 | 16 | 29 | 55.172 |
|  |  | N-isopropylammelide | AOO | 3 | 1 | 0.657 | 3lsc(A) | 30.3 | 4.9 | 16 | 29 | 55.172 |
|  |  | ethylamine | HAE | 18 | 1 | 0.294 | 1fwe(C) | 36.8 | 4.2 | 5 | 5 | 100 |
| 2.4.1.155 | P97259 | UDP | HEC | 1 | 1 | 0.41 | 2ozy(A) | 27.8 | 8.9 | 16 | 24 | 66.667 |
|  |  | UDP-N-acetyl-alpha-D-glucosamine | HEC | 1 | 1 | 0.499 | 2ozy(A) | 27.8 | 8.9 | 16 | 24 | 66.667 |
| 1.14.13.73 | P98183 | tabersonine | HEM-JKF | 1 | 17 | 0.454 | 3tik(A) | 24.2 | 9.70E-12 | 40 | 67 | 59.701 |
|  |  | 16-hydroxytabersonine | HEM-JKF | 1 | 17 | 0.473 | 3tik(A) | 24.2 | 9.70E-12 | 40 | 67 | 59.701 |
|  |  | NADPH | HEM-KLN | 1 | 16 | 0.575 | 2v0m(A) | 26.8 | 7.40E-24 | 40 | 73 | 54.795 |
|  |  | NADP(+) | HEM-KLN | 1 | 16 | 0.558 | 2v0m(A) | 26.8 | 7.40E-24 | 40 | 73 | 54.795 |
| 4.2.3.28 | Q00G37 | diphosphoric acid | POP | 2 | 1 | 0.923 | 1n23(A) | 22.2 | 2.40E-10 | 24 | 30 | 80 |
|  |  | diphosphate(4-) | POP | 2 | 1 | 1 | 1n23(A) | 22.2 | 2.40E-10 | 24 | 30 | 80 |
|  |  | 5beta,9alpha,10alpha-labda-8(20),13-dien-15-yl diphosphate | FFF | 1 | 4 | 0.497 | 5eau(A) | 20.6 | 3.50E-11 | 7 | 26 | 26.923 |
| 2.3.1.111 | Q02251 | NADPH | NDP | 1 | 1 | 1 | 3mjs(A) | 38.9 | 2.00E-22 | 47 | 66 | 71.212 |
|  |  | coenzyme A | COZ | 1 | 7 | 1 | 2g2z(A) | 28.1 | 0.14 | 17 | 32 | 53.125 |
|  |  | NADP(+) | NAP | 1 | 2 | 0.998 | 2vz9(A) | 35.9 | 9.40E-21 | 44 | 59 | 74.576 |
|  |  | methylmalonyl-CoA | CO8 | 1 | 14 | 0.973 | 4a0s(D) | 26.8 | 37 | 5 | 19 | 26.316 |
| 1.2.1.50 | Q03324 | NADPH | 15P | 20 | 2 | 0.022 | 1u0m(A) | 26.5 | 13 | 1 | 4 | 25 |
|  |  | coenzyme A | 15P | 20 | 2 | 0.025 | 1u0m(A) | 26.5 | 13 | 1 | 4 | 25 |
|  |  | NADP(+) | 15P | 20 | 2 | 0.023 | 1u0m(A) | 26.5 | 13 | 1 | 4 | 25 |
| 1.14.12.7 | Q05183 | phthalic acid | PRO | 14 | 1 | 0.223 | 3vca(A) | 24.6 | 0.0052 | 3 | 10 | 30 |
|  |  | NADH | OXZ-BGC-BGC | 3 | 1 | 0.356 | 2yjq(A) | 23.2 | 43 | 9 | 22 | 40.909 |
|  |  | NAD(+) | OXZ-BGC-BGC | 3 | 1 | 0.364 | 2yjq(A) | 23.2 | 43 | 9 | 22 | 40.909 |
|  |  | cis-4,5-dihydroxycyclohexa-2,6-diene-1,2-dicarboxylic acid | PRO | 14 | 1 | 0.191 | 3vca(A) | 24.6 | 0.0052 | 3 | 10 | 30 |
| 3.1.1.79 | Q05469 | glycerol | GOL | 21 | 2 | 1 | 2y1k(A) | 28.7 | 33 | 9 | 13 | 69.231 |
| 2.3.1.19 | Q05624 | coenzyme A | COA | 1 | 2 | 1.000 | 2af4(C) | 27.6 | 2.90E-08 | 18 | 44 | 40.909 |
|  |  | butyryl-CoA | COA | 1 | 2 | 0.964 | 2af4(C) | 27.6 | 2.90E-08 | 18 | 44 | 40.909 |
|  |  | butanoyl dihydrogen phosphate | UVW | 4 | 1 | 0.667 | 1xco(A) | 26.3 | 1.50E-05 | 11 | 13 | 84.615 |
|  |  | phosphoric acid | PO4 | 12 | 1 | 0.857 | 1yco(A) | 38.8 | 4.80E-28 | 8 | 8 | 100 |
| 2.7.8.29 | Q08D11 | L-serine | GLP | 2 | 1 | 0.066 | 3cxq(A) | 27.4 | 19 | 11 | 17 | 64.706 |
|  |  | ethanolamine | PO4 | 7 | 1 | 0.067 | 2oaq(1) | 25.8 | 6.2 | 3 | 15 | 20 |
| 2.4.1.143 | Q09326 | UDP | ADP | 1 | 13 | 0.565 | 1rk2(A) | 36.5 | 17 | 2 | 27 | 7.407 |
|  |  | UDP-N-acetyl-alpha-D-glucosamine | GDP-AF3 | 1 | 8 | 0.541 | 1he1(C) | 22.9 | 8.1 | 21 | 56 | 37.5 |
| 2.4.1.144 | Q09327 | UDP | PGE | 3 | 2 | 0.034 | 4dwh(C) | 31.5 | 0.5 | 1 | 4 | 25 |
|  |  | UDP-N-acetyl-alpha-D-glucosamine | PGE | 3 | 2 | 0.026 | 4dwh(C) | 31.5 | 0.5 | 1 | 4 | 25 |
| 2.4.1.46 | Q0DWQ1 | UDP | UD1 | 1 | 1 | 0.769 | 3s2u(A) | 25.9 | 0.19 | 19 | 32 | 59.375 |
|  |  | UDP-D-galactose | UD1 | 1 | 1 | 0.787 | 3s2u(A) | 25.9 | 0.19 | 19 | 32 | 59.375 |
| 3.6.3.29 | Q0HYN8 | ATP | ATP | 1 | 3 | 1 | 2pze(A) | 24.3 | 3.80E-08 | 28 | 33 | 84.848 |
|  |  | ADP | ADP-BEF | 1 | 2 | 1 | 3pux(A) | 34.6 | 3.40E-24 | 30 | 41 | 73.171 |
|  |  | phosphoric acid | \_PI | 2 | 2 | 0.857 | 3tif(A) | 27.4 | 1.00E-06 | 13 | 14 | 92.857 |
|  |  | molybdate | MOO | 16 | 1 | 1 | 1h9m(A) | 29.3 | 3.7 | 5 | 8 | 62.5 |
| 4.1.3.32 | Q0QLE4 | (2R,3S)-2,3-dimethylmalic acid | SIN | 1 | 3 | 0.809 | 1f8i(A) | 24.1 | 0.00013 | 18 | 30 | 60 |
|  |  | propionic acid | PYR | 2 | 2 | 0.786 | 1mzx(A) | 37.2 | 1.20E-36 | 15 | 19 | 78.947 |
|  |  | pyruvic acid | PYR | 2 | 2 | 1 | 1mzx(A) | 37.2 | 1.20E-36 | 15 | 19 | 78.947 |
| 2.5.1.77 | Q0W6V4 | S-adenosyl-L-methionine | SAM | 6 | 1 | 0.998 | 1r30(A) | 19.1 | 19 | 13 | 34 | 38.235 |
|  |  | 5'-deoxyadenosine | 5AD | 6 | 2 | 1 | 3iix(A) | 26.4 | 2.00E-05 | 12 | 24 | 50 |
|  |  | oxalic acid | CO3 | 18 | 2 | 0.5 | 3iiz(A) | 26.4 | 2.00E-05 | 1 | 3 | 33.333 |
|  |  | L-methionine | TRS | 24 | 2 | 0.314 | 1pvn(A) | 23.3 | 47 | 2 | 7 | 28.571 |
|  |  | (4-hydroxyphenyl)pyruvic acid | CPS | 21 | 1 | 0.211 | 3iix(A) | 26.4 | 2.00E-05 | 5 | 9 | 55.556 |
|  |  | 5-amino-6-(D-ribitylamino)uracil | 5AD | 6 | 2 | 0.246 | 3iix(A) | 26.4 | 2.00E-05 | 12 | 24 | 50 |
| 1.3.1.54 | Q10680 | NADPH | 5CA | 1 | 1 | 0.641 | 1nj1(A) | 45.2 | 5 | 15 | 51 | 29.412 |
|  |  | NADP(+) | 5CA | 1 | 1 | 0.653 | 1nj1(A) | 45.2 | 5 | 15 | 51 | 29.412 |
|  |  | precorrin-6X | P5A | 1 | 3 | 0.406 | 1nj5(A) | 45.2 | 5 | 15 | 51 | 29.412 |
|  |  | precorrin-6Y | P5A | 1 | 3 | 0.394 | 1nj5(A) | 45.2 | 5 | 15 | 51 | 29.412 |
| 4.4.1.9 | Q1KLZ1 | L-cysteine | DSN | 15 | 1 | 0.68 | 4d9n(A) | 35.7 | 2.6 | 7 | 13 | 53.846 |
|  |  | 3-cyano-L-alanine | DSN | 15 | 1 | 0.531 | 4d9n(A) | 35.7 | 2.6 | 7 | 13 | 53.846 |
| 1.11.1.20 | Q28IJ3 | prostaglandin F2alpha | PRO-GLY-ALA-TYR-ASP | 3 | 1 | 0.2 | 1gmd(B) | 29.9 | 29 | 9 | 38 | 23.684 |
|  |  | prostaglandin H2 | PRO-GLY-ALA-TYR-ASP | 3 | 1 | 0.211 | 1gmd(B) | 29.9 | 29 | 9 | 38 | 23.684 |
| 4.2.3.20 | Q2XSC6 | diphosphoric acid | POP | 3 | 1 | 0.923 | 1n23(A) | 63 | 1.60E-99 | 30 | 30 | 100 |
|  |  | diphosphate(4-) | POP | 3 | 1 | 1 | 1n23(A) | 63 | 1.60E-99 | 30 | 30 | 100 |
|  |  | geranyl diphosphate | 0FV | 5 | 5 | 0.821 | 3v1x(A) | 22.2 | 9.2 | 15 | 34 | 44.118 |
| 2.1.1.143 | Q39227 | S-adenosyl-L-methionine | SAM | 1 | 2 | 1 | 2fk8(A) | 24.8 | 0.0011 | 30 | 48 | 62.5 |
|  |  | S-adenosyl-L-homocysteine | SAH | 1 | 1 | 1 | 3vc2(A) | 22.6 | 3.20E-07 | 31 | 51 | 60.784 |
|  |  | 24-methylidenelophenol | ASE/a> | 32 | 1 | 0.25 | 3t7r(A) | 26.4 | 0.0069 | 0 | 2 | 0 |
| 2.1.1.117 | Q39522 | S-adenosyl-L-methionine | SAH | 1 | 1 | 1 | 3reo(A) | 48.2 | 2.00E-49 | 40 | 42 | 95.238 |
|  |  | S-adenosyl-L-homocysteine | SAH | 1 | 1 | 1 | 3reo(A) | 48.2 | 2.00E-49 | 40 | 42 | 95.238 |
|  |  | (S)-scoulerine | ASE | 5 | 1 | 0.324 | 4a6e(A) | 24.2 | 1.20E-06 | 13 | 28 | 46.429 |
|  |  | (S)-tetrahydrocolumbamine | HMK | 10 | 2 | 0.313 | 1zga(A) | 35.9 | 2.30E-20 | 10 | 25 | 40 |
| 3.1.3.78 | Q3TWL2 | phosphoric acid | NAG-NAG-MAN | 3 | 1 | 0.005 | 3bl8(A) | 23.9 | 29 | 1 | 6 | 16.667 |
| 2.1.1.60 | Q3U2J5 | S-adenosyl-L-methionine | SAM | 1 | 3 | 1 | 3uj6(A) | 21.1 | 35 | 15 | 40 | 37.5 |
|  |  | S-adenosyl-L-homocysteine | SAH | 1 | 1 | 1 | 4fgz(A) | 21.1 | 35 | 16 | 43 | 37.209 |
| 2.3.1.78 | Q3UDW8 | coenzyme A | NAG-NAG | 6 | 1 | 0.353 | 1mn2(A) | 22.8 | 10 | 7 | 14 | 50 |
| 2.3.1.88 | Q3UX61 | coenzyme A | COA | 1 | 2 | 1 | 2x7b(A) | 38.3 | 1.20E-17 | 32 | 46 | 69.565 |
|  |  | acetyl-CoA | ACO | 1 | 1 | 1 | 2ob0(C) | 22.9 | 0.00038 | 32 | 53 | 60.377 |
| 1.14.11.25 | Q40061 | 2-oxoglutaric acid | AKG | 3 | 1 | 1 | 2brt(A) | 30 | 2.40E-31 | 25 | 25 | 100 |
|  |  | succinic acid | SIN | 3 | 3 | 1 | 1gp6(A) | 30 | 2.40E-31 | 16 | 17 | 94.118 |
|  |  | mugineic acid | SCV | 5 | 7 | 0.207 | 1hb3(A) | 23 | 0.0013 | 20 | 39 | 51.282 |
| 1.14.11.24 | Q40062 | 2-oxoglutaric acid | AKG | 1 | 1 | 1 | 2brt(A) | 29.1 | 3.60E-26 | 25 | 25 | 100 |
|  |  | succinic acid | SIN | 1 | 5 | 1 | 1gp6(A) | 29.1 | 3.60E-26 | 15 | 17 | 88.235 |
|  |  | mugineic acid | PNN | 2 | 2 | 0.18 | 1uof(A) | 22.5 | 4.1 | 14 | 23 | 60.87 |
| 1.1.99.32 | Q47944 | L-sorbopyranose | MAN | 4 | 2 | 0.965 | 1kdg(A) | 28.6 | 0.013 | 3 | 7 | 42.857 |
| 1.5.8.1 | Q48303 | dimethylamine | BTB | 14 | 2 | 0.222 | 3gr8(A) | 26.3 | 1.20E-10 | 8 | 19 | 42.105 |
| 2.3.1.59 | Q52424 | coenzyme A | COA | 1 | 2 | 1 | 3ld2(A) | 30.4 | 21 | 26 | 47 | 55.319 |
|  |  | acetyl-CoA | ACO | 1 | 1 | 1 | 2bei(A) | 29.8 | 12 | 33 | 51 | 64.706 |
|  |  | gentamycin C1a | TOY | 2 | 2 | 0.832 | 1m4d(A) | 32.9 | 1.30E-23 | 26 | 32 | 81.25 |
|  |  | N(2')-acetylgentamycin C1a | TOY | 2 | 2 | 0.709 | 1m4d(A) | 32.9 | 1.30E-23 | 26 | 32 | 81.25 |
| 4.1.1.82 | Q54271 | 3-phosphonopyruvic acid | RMN | 15 | 2 | 0.191 | 1mcz(A) | 29.7 | 0.00035 | 3 | 6 | 50 |
|  |  | phosphonoacetaldehyde | PO4 | 7 | 1 | 0.261 | 1ozg(A) | 33.3 | 0.0019 | 8 | 10 | 80 |
| 2.3.1.23 | Q54DX7 | coenzyme A | ZS0-HEM | 3 | 2 | 0.491 | 3zs0(C) | 23.8 | 3.3 | 5 | 18 | 27.778 |
| 2.1.1.205 | Q54VA8 | S-adenosyl-L-methionine | SAM | 1 | 1 | 1 | 2nyu(A) | 36.7 | 6.40E-20 | 32 | 43 | 74.419 |
|  |  | S-adenosyl-L-homocysteine | SAH | 1 | 2 | 1 | 3reo(A) | 24.4 | 10 | 16 | 42 | 38.095 |
| 3.1.3.69 | Q55034 | 2-O-(beta-D-glucosyl)-sn-glycerol | NAG | 13 | 1 | 0.41 | 1cf3(A) | 28.9 | 3.9 | 3 | 9 | 33.333 |
|  |  | phosphoric acid | PEG | 15 | 1 | 0.059 | 3qvp(A) | 28.9 | 3.9 | 3 | 6 | 50 |
| 4.4.1.23 | Q56837 | coenzyme M | SO4 | 3 | 1 | 0.389 | 1u1h(A) | 21.8 | 0.39 | 7 | 12 | 58.333 |
|  |  | (S)-2-hydroxypropyl-CoM | MET | 1 | 3 | 0.213 | 1u1j(A) | 21.5 | 3.7 | 15 | 22 | 68.182 |
|  |  | (R)-2-hydroxypropyl-CoM | MET | 1 | 3 | 0.213 | 1u1j(A) | 21.5 | 3.7 | 15 | 22 | 68.182 |
|  |  | (S)-1,2-epoxypropane | MRY | 6 | 2 | 0.364 | 1xdj(A) | 26.4 | 0.53 | -1 | 2 | -50 |
| 1.1.1.269 | Q56841 | NADH | NAI | 1 | 4 | 1 | 1nfq(A) | 37.2 | 4.70E-19 | 46 | 63 | 73.016 |
|  |  | NAD(+) | NAD | 1 | 1 | 0.998 | 2wsb(A) | 50.2 | 2.40E-37 | 54 | 67 | 80.597 |
|  |  | 2-oxopropyl-CoM | KPC | 11 | 1 | 1 | 2cfc(A) | 41.1 | 7.80E-31 | 13 | 23 | 56.522 |
|  |  | (S)-2-hydroxypropyl-CoM | KPC | 11 | 1 | 0.82 | 2cfc(A) | 41.1 | 7.80E-31 | 13 | 23 | 56.522 |
| 1.8.98.1 | Q58153 | coenzyme B | COD | 3 | 3 | 0.168 | 3s89(A) | 29.3 | 3.5 | 5 | 15 | 33.333 |
|  |  | dihydromethanophenazine | COA | 3 | 2 | 0.223 | 3qov(A) | 29.3 | 3.5 | 5 | 24 | 20.833 |
| 1.1.5.8 | Q59086 | 3-dehydroquinic acid | TFB | 6 | 1 | 0.33 | 1kb0(A) | 27.5 | 2.10E-10 | 7 | 14 | 50 |
|  |  | (-)-quinic acid | TFB | 6 | 1 | 0.341 | 1kb0(A) | 27.5 | 2.10E-10 | 7 | 14 | 50 |
|  |  | (-)-quinate | TFB | 6 | 1 | 0.337 | 1kb0(A) | 27.5 | 2.10E-10 | 7 | 14 | 50 |
|  |  | 3-dehydroquinate | TFB | 6 | 1 | 0.326 | 1kb0(A) | 27.5 | 2.10E-10 | 7 | 14 | 50 |
|  |  | 1,4-benzoquinone | ACN | 11 | 1 | 0.212 | 1kv9(A) | 26.2 | 0.011 | 5 | 10 | 50 |
|  |  | hydroquinone | ACN | 11 | 1 | 0.15 | 1kv9(A) | 26.2 | 0.011 | 5 | 10 | 50 |
| 2.1.1.140 | Q5C9L6 | S-adenosyl-L-methionine | SAH | 1 | 1 | 1 | 1kpg(A) | 26.1 | 1.10E-11 | 32 | 54 | 59.259 |
|  |  | S-adenosyl-L-homocysteine | SAH | 1 | 1 | 1 | 1kpg(A) | 26.1 | 1.10E-11 | 32 | 54 | 59.259 |
|  |  | (S)-N-methylcoclaurine | 849 | 17 | 1 | 0.289 | 2y1w(A) | 24.5 | 0.29 | 6 | 27 | 22.222 |
|  |  | (S)-coclaurine | CQA | 24 | 1 | 0.287 | 4fgz(A) | 24.2 | 0.0025 | 4 | 14 | 28.571 |
| 4.4.1.17 | Q5F339 | ferroheme b | ACI-G6D-GLC-ACI-G6D-BGC | 1 | 1 | 0.357 | 4e2o(A) | 23.9 | 4.6 | 14 | 39 | 35.897 |
| 2.3.1.93 | Q5H873 | coenzyme A | MLC | 1 | 1 | 0.952 | 2e1t(A) | 31.8 | 0.0051 | 21 | 49 | 42.857 |
|  |  | 2-methylcrotonoyl-CoA | MLC | 1 | 1 | 0.971 | 2e1t(A) | 31.8 | 0.0051 | 21 | 49 | 42.857 |
|  |  | 13-hydroxylupanine | MLC | 1 | 1 | 0.192 | 2e1t(A) | 31.8 | 0.0051 | 21 | 49 | 42.857 |
| 2.7.8.5 | Q5HPQ8 | CMP | BOG | 1 | 1 | 0.224 | 1c4o(A) | 31.1 | 14 | 1 | 6 | 16.667 |
|  |  | sn-glycerol 3-phosphate | BOG | 1 | 1 | 0.064 | 1c4o(A) | 31.1 | 14 | 1 | 6 | 16.667 |
| 2.4.1.254 | Q5NTH0 | UDP | UDP | 1 | 3 | 1 | 2acv(A) | 38.1 | 6.50E-24 | 29 | 37 | 78.378 |
|  |  | UDP-alpha-D-glucuronic acid | UPG | 1 | 1 | 0.979 | 2acw(A) | 29 | 0.053 | 34 | 53 | 64.151 |
|  |  | cyanidin 3-O-beta-D-glucoside | MYC | 10 | 2 | 0.476 | 3hbf(A) | 37.7 | 6.00E-20 | 2 | 6 | 33.333 |
| 2.8.2.21 | Q5RJQ0 | adenosine 3',5'-bismonophosphate | A3P | 2 | 1 | 1 | 4gox(A) | 29.5 | 0.012 | 15 | 54 | 27.778 |
|  |  | 3'-phospho-5'-adenylyl sulfate | A3P | 2 | 1 | 0.953 | 4gox(A) | 29.5 | 0.012 | 15 | 54 | 27.778 |
| 2.5.1.91 | Q5U2R1 | 2-trans,6-trans-farnesyl diphosphate | FPP | 3 | 1 | 1 | 3aq0(A) | 29.4 | 6.30E-20 | 13 | 25 | 52 |
|  |  | all-trans-decaprenyl diphosphate | FPP | 3 | 1 | 1 | 3aq0(A) | 29.4 | 6.30E-20 | 13 | 25 | 52 |
|  |  | diphosphoric acid | PPV | 5 | 1 | 1 | 3aq0(F) | 28.5 | 5.30E-15 | 7 | 8 | 87.5 |
|  |  | diphosphate(4-) | DPO | 5 | 3 | 1 | 1rqi(A) | 22.3 | 27 | 2 | 17 | 11.765 |
|  |  | isopentenyl diphosphate | IPE | 4 | 2 | 1 | 3oab(D) | 29.7 | 0.36 | 9 | 22 | 40.909 |
| 5.3.3.5 | Q60490 | 5alpha-cholest-7-en-3beta-ol | HEA | 1 | 1 | 0.176 | 3oma(A) | 28.8 | 3 | 17 | 56 | 30.357 |
|  |  | 5alpha-cholest-8-en-3beta-ol | HEA | 1 | 1 | 0.174 | 3oma(A) | 28.8 | 3 | 17 | 56 | 30.357 |
| 2.3.1.26 | Q61263 | coenzyme A | ANP | 1 | 1 | 0.831 | 3mfu(A) | 25.5 | 44 | 8 | 33 | 24.242 |
|  |  | cholesterol | PQE | 4 | 1 | 0.167 | 2nr9(A) | 19.4 | 20 | 3 | 21 | 14.286 |
| 3.6.1.27 | Q65FP0 | phosphoric acid | BCN | 1 | 1 | 0.037 | 3gia(A) | 23.5 | 21 | 2 | 2 | 100 |
|  |  | ditrans,polycis-undecaprenyl phosphate | D10 | 2 | 1 | 0.12 | 3gia(A) | 23.5 | 21 | 1 | 6 | 16.667 |
|  |  | di-trans,poly-cis-undecaprenyl diphosphate | D10 | 2 | 1 | 0.094 | 3gia(A) | 23.5 | 21 | 1 | 6 | 16.667 |
| 2.1.1.62 | Q6EU10 | S-adenosyl-L-methionine | NAD | 1 | 1 | 0.662 | 2dc1(A) | 26.5 | 10 | 19 | 50 | 38 |
|  |  | S-adenosyl-L-homocysteine | NAD | 1 | 1 | 0.661 | 2dc1(A) | 26.5 | 10 | 19 | 50 | 38 |
| 1.14.13.117 | Q6J541 | NADPH | HEM-X2N | 1 | 9 | 0.568 | 2x2n(A) | 21.1 | 9.90E-05 | 31 | 72 | 43.056 |
|  |  | NADP(+) | HEM-X2N | 1 | 9 | 0.544 | 2x2n(A) | 21.1 | 9.90E-05 | 31 | 72 | 43.056 |
|  |  | L-isoleucine | TAM | 36 | 2 | 0.555 | 3uas(A) | 23.3 | 1.70E-14 | 4 | 13 | 30.769 |
| 2.1.1.112 | Q6NMK1 | S-adenosyl-L-methionine | EDO | 2 | 1 | 0.013 | 2g0t(A) | 23.9 | 4.6 | 4 | 12 | 33.333 |
|  |  | S-adenosyl-L-homocysteine | PO4 | 1 | 1 | 0.013 | 2g0t(A) | 23.9 | 4.6 | 10 | 15 | 66.667 |
| 2.1.1.88 | Q6VMW0 | S-adenosyl-L-methionine | SAM | 1 | 2 | 1 | 3i5u(A) | 27.8 | 9.10E-07 | 30 | 42 | 71.429 |
|  |  | S-adenosyl-L-homocysteine | SAH | 1 | 1 | 1 | 3reo(A) | 37 | 1.70E-31 | 36 | 42 | 85.714 |
| 1.14.99.37 | Q6WG30 | taxa-4(20),11-dien-5alpha-ol | ASD | 18 | 1 | 0.646 | 3s79(A) | 22.5 | 1.10E-08 | 9 | 21 | 42.857 |
|  |  | taxa-4,11-diene | EL3 | 55 | 1 | 0.8 | 3el3(A) | 23.2 | 5.40E-09 | 4 | 10 | 40 |
| 4.2.3.30 | Q6Z5J6 | diphosphoric acid | POP | 1 | 1 | 0.923 | 1n23(A) | 23.9 | 7.60E-12 | 26 | 30 | 86.667 |
|  |  | diphosphate(4-) | POP | 1 | 1 | 1 | 1n23(A) | 23.9 | 7.60E-12 | 26 | 30 | 86.667 |
|  |  | 5beta,9alpha,10alpha-labda-8(20),13-dien-15-yl diphosphate | FPP | 2 | 6 | 0.556 | 1jcq(B) | 37.5 | 30 | 5 | 16 | 31.25 |
| 1.16.1.7 | Q75CQ8 | NADH | ADP | 3 | 9 | 0.817 | 2q2r(A) | 35 | 0.028 | -5 | 17 | -29.412 |
|  |  | NAD(+) | ADP | 3 | 9 | 0.853 | 2q2r(A) | 35 | 0.028 | -5 | 17 | -29.412 |
| 4.1.2.34 | Q79EM8 | pyruvic acid | PYR | 6 | 3 | 1 | 3di1(A) | 28.7 | 5.80E-07 | 10 | 18 | 55.556 |
|  |  | 2-formylbenzoic acid | OXL | 6 | 2 | 0.238 | 3u8g(A) | 30.6 | 2.10E-08 | 12 | 18 | 66.667 |
|  |  | (3Z)-4-(2-carboxyphenyl)-2-oxobut-3-enoic acid | RSH | 7 | 2 | 0.144 | 3nev(A) | 27.2 | 2.60E-09 | 12 | 33 | 36.364 |
| 2.1.1.175 | Q7F8T6 | S-adenosyl-L-methionine | SAM | 1 | 11 | 1 | 2zvj(A) | 21.7 | 0.042 | 29 | 53 | 54.717 |
|  |  | S-adenosyl-L-homocysteine | SAH | 1 | 2 | 1 | 1sui(A) | 54.9 | 2.10E-59 | 34 | 42 | 80.952 |
|  |  | tricetin | KOM | 5 | 1 | 0.357 | 2zvj(A) | 21.7 | 0.042 | 15 | 23 | 65.217 |
| 6.3.1.14 | Q7L8W6 | AMP | AMP | 1 | 2 | 1 | 3rk0(A) | 33.6 | 0.02 | 25 | 29 | 86.207 |
|  |  | ATP | ATP | 1 | 1 | 1 | 3rk1(A) | 35.1 | 0.092 | 32 | 42 | 76.19 |
|  |  | diphosphoric acid | PO4 | 2 | 1 | 0.461 | 3rk1(A) | 35.1 | 0.05 | 10 | 14 | 71.429 |
|  |  | diphosphate(4-) | PO4 | 2 | 1 | 0.538 | 3rk1(A) | 35.1 | 0.05 | 10 | 14 | 71.429 |
| 3.6.3.31 | Q7MKU3 | ATP | ATP | 1 | 4 | 1 | 2r6g(A) | 45.1 | 2.70E-42 | 27 | 33 | 81.818 |
|  |  | ADP | ADP | 1 | 7 | 1 | 2pmk(A) | 34.1 | 9.70E-22 | 23 | 37 | 62.162 |
|  |  | phosphoric acid | \_PI | 5 | 2 | 0.857 | 3tif(A) | 36.1 | 1.60E-15 | 13 | 14 | 92.857 |
| 4.1.2.44 | Q84HH6 | formic acid | BCT | 14 | 1 | 0.857 | 3h02(F) | 21.9 | 13 | 8 | 19 | 42.105 |
|  |  | formate | BCT | 14 | 1 | 1 | 3h02(F) | 21.9 | 13 | 8 | 19 | 42.105 |
| 1.5.3.13 | Q865R1 | spermidine | SPD | 17 | 6 | 1 | 3l1r(A) | 43.2 | 1.90E-07 | 1 | 17 | 5.882 |
|  |  | N(1)-acetylspermine | SP5 | 17 | 1 | 1 | 3cnd(B) | 38.2 | 0.00031 | 4 | 21 | 19.048 |
|  |  | N(1)-acetylspermidine | SP7 | 17 | 3 | 0.979 | 3cnp(B) | 38.2 | 0.00068 | 2 | 20 | 10 |
|  |  | putrescine | DIA | 14 | 2 | 0.5 | 3bi5(A) | 36.6 | 0.00027 | 1 | 12 | 8.333 |
| 2.6.1.76 | Q8ESU8 | L-2,4-diaminobutyric acid | HOZ | 7 | 1 | 0.724 | 2hp1(B) | 39.1 | 0.015 | 14 | 21 | 66.667 |
|  |  | L-glutamic acid | HOZ | 7 | 1 | 0.742 | 2hp1(B) | 39.1 | 0.015 | 14 | 21 | 66.667 |
|  |  | L-aspartic 4-semialdehyde | SSN | 10 | 2 | 0.692 | 3q8n(A) | 30.5 | 8.10E-16 | 8 | 13 | 61.538 |
|  |  | L-aspartate 4-semialdehyde | SSN | 10 | 2 | 0.667 | 3q8n(A) | 30.5 | 8.10E-16 | 8 | 13 | 61.538 |
|  |  | 2-oxoglutaric acid | AKG | 10 | 1 | 1 | 2cjh(A) | 27.3 | 0.017 | 12 | 18 | 66.667 |
| 1.14.12.22 | Q8G8B6 | NADPH | OCH | 2 | 1 | 0.147 | 1z03(A) | 42.6 | 1.90E-23 | 15 | 21 | 71.429 |
|  |  | NADH | OCH | 2 | 1 | 0.149 | 1z03(A) | 42.6 | 1.90E-23 | 15 | 21 | 71.429 |
|  |  | NADP(+) | OCH | 2 | 1 | 0.143 | 1z03(A) | 42.6 | 1.90E-23 | 15 | 21 | 71.429 |
|  |  | NAD(+) | OCH | 2 | 1 | 0.145 | 1z03(A) | 42.6 | 1.90E-23 | 15 | 21 | 71.429 |
|  |  | 2'-aminobiphenyl-2,3-diol | OCH | 2 | 1 | 0.19 | 1z03(A) | 42.6 | 1.90E-23 | 15 | 21 | 71.429 |
| 2.6.1.86 | Q8GMH4 | chorismic acid | ISC | 1 | 1 | 0.663 | 3hwo(A) | 30 | 3.20E-14 | 32 | 40 | 80 |
|  |  | L-glutamine | GLU | 13 | 1 | 0.823 | 1i7q(B) | 32.3 | 9.50E-23 | 3 | 3 | 100 |
|  |  | L-glutamic acid | GLU | 13 | 1 | 1 | 1i7q(B) | 32.3 | 9.50E-23 | 3 | 3 | 100 |
| 2.4.1.230 | Q8L163 | D-glucopyranose | ATP | 1 | 17 | 0.174 | 3hmn(A) | 37.5 | 47 | 2 | 4 | 50 |
|  |  | beta-D-glucose 1-phosphate | ATP | 1 | 17 | 0.251 | 3hmn(A) | 37.5 | 47 | 2 | 4 | 50 |
|  |  | phosphoric acid | IPA | 14 | 2 | 0.091 | 3hmp(A) | 37.5 | 47 | 3 | 7 | 42.857 |
| 3.3.2.9 | Q8MZR6 | (R,R)-hydrobenzoin | BEZ | 12 | 1 | 0.404 | 4f5z(A) | 25.4 | 0.29 | 9 | 18 | 50 |
| 2.7.1.52 | Q8N0W3 | ATP | ATP | 1 | 2 | 1 | 1kvk(A) | 41.7 | 5.3 | 14 | 44 | 31.818 |
|  |  | ADP | ADP | 1 | 8 | 1 | 1w0k(A) | 46.7 | 28 | 6 | 20 | 30 |
|  |  | L-fucopyranose | GLA | 6 | 1 | 0.882 | 1s4e(G) | 26.5 | 3.3 | 5 | 7 | 71.429 |
|  |  | L-fucopyranose 1-phosphate | R1P | 26 | 1 | 0.695 | 3qpb(A) | 38.8 | 8.2 | -3 | 28 | -10.714 |
| 2.4.1.187 | Q8NXS7 | UDP | UDP | 5 | 1 | 1 | 3otk(A) | 26.3 | 38 | -6 | 28 | -21.429 |
|  |  | UDP-N-acetyl-alpha-D-mannosamine | UDP | 5 | 1 | 0.673 | 3otk(A) | 26.3 | 38 | -6 | 28 | -21.429 |
|  |  | N-acetyl-D-glucosaminyl-1-diphospho-ditrans,polycis-undecaprenol | GAL-NGA | 1 | 1 | 0.491 | 2gam(A) | 26.3 | 37 | 9 | 34 | 26.471 |
| 2.5.1.42 | Q8PV96 | 2-trans,6-trans,10-trans-geranylgeranyl diphosphate | BNG | 1 | 1 | 0.123 | 4dxw(A) | 29.6 | 2.4 | 4 | 11 | 36.364 |
|  |  | diphosphoric acid | BNG | 1 | 1 | 0.009 | 4dxw(A) | 29.6 | 2.4 | 4 | 11 | 36.364 |
|  |  | diphosphate(4-) | BNG | 1 | 1 | 0.009 | 4dxw(A) | 29.6 | 2.4 | 4 | 11 | 36.364 |
|  |  | sn-3-O-(geranylgeranyl)glycerol 1-phosphate | BNG | 1 | 1 | 0.2 | 4dxw(A) | 29.6 | 2.4 | 4 | 11 | 36.364 |
| 3.5.1.90 | Q8Q0G3 | (2R)-1-aminopropan-2-ol | SO4 | 7 | 1 | 0.118 | 4frf(A) | 21.9 | 44 | 5 | 11 | 45.455 |
|  |  | adenosylcobyric acid | SO4 | 7 | 1 | 0.008 | 4frf(A) | 21.9 | 44 | 5 | 11 | 45.455 |
| 2.7.8.14 | Q8RKJ2 | CMP | HEM | 1 | 1 | 0.235 | 1chj(A) | 33.3 | 37 | 20 | 55 | 36.364 |
|  |  | CDP-ribitol | HEM | 1 | 1 | 0.232 | 1chj(A) | 33.3 | 37 | 20 | 55 | 36.364 |
| 2.7.7.69 | Q8RWE8 | GDP | PYR | 15 | 2 | 0.017 | 3di1(A) | 24.8 | 1.1 | 2 | 18 | 11.111 |
|  |  | GDP-L-galactose | PYR | 15 | 2 | 0.019 | 3di1(A) | 24.8 | 1.1 | 2 | 18 | 11.111 |
|  |  | phosphoric acid | GOL | 4 | 2 | 0.077 | 1ur9(A) | 23.5 | 5.3 | 8 | 16 | 50 |
| 1.14.13.122 | Q8S7E1 | NADPH | SMA | 13 | 1 | 0.308 | 2ybb(c) | 28.1 | 1.6 | 6 | 8 | 75 |
|  |  | NADP(+) | NGO | 5 | 1 | 0.315 | 2wzh(A) | 28.8 | 21 | 10 | 37 | 27.027 |
|  |  | chlorophyllide a | SMA | 13 | 1 | 0.324 | 2ybb(c) | 28.1 | 1.6 | 6 | 8 | 75 |
| 2.3.1.25 | Q8S8S2 | coenzyme A | KAR | 32 | 1 | 0.496 | 1xa5(A) | 35 | 0.034 | 2 | 16 | 12.5 |
| 2.4.1.229 | Q8T1C6 | UDP | SAL | 9 | 1 | 0.073 | 2y7k(A) | 26.9 | 38 | 3 | 5 | 60 |
|  |  | UDP-N-acetyl-alpha-D-glucosamine | SAL | 9 | 1 | 0.062 | 2y7k(A) | 26.9 | 38 | 3 | 5 | 60 |
| 2.8.3.6 | Q8VPF3 | 3-oxoadipic acid | FLC | 4 | 1 | 0.628 | 1b0m(A) | 30.1 | 14 | 10 | 15 | 66.667 |
|  |  | succinic acid | FLC | 4 | 1 | 0.643 | 1b0m(A) | 30.1 | 14 | 10 | 15 | 66.667 |
|  |  | 3-oxoadipate(2-) | FLC | 4 | 1 | 0.657 | 1b0m(A) | 30.1 | 14 | 10 | 15 | 66.667 |
|  |  | succinyl-CoA | SF4-ATH | 1 | 3 | 0.366 | 1fgh(A) | 30.1 | 3 | 14 | 19 | 73.684 |
|  |  | 3-oxoadipyl-CoA | SF4-NIC | 1 | 2 | 0.366 | 8acn(A) | 30.1 | 14 | 14 | 19 | 73.684 |
| 2.7.1.89 | Q8X8G6 | ATP | FAD | 1 | 5 | 0.673 | 1zp0(A) | 31.7 | 26 | 0 | 2 | 0 |
|  |  | ADP | FAD | 1 | 5 | 0.677 | 1zp0(A) | 31.7 | 26 | 0 | 2 | 0 |
|  |  | thiamine(1+) | FAD | 1 | 5 | 0.179 | 1zp0(A) | 31.7 | 26 | 0 | 2 | 0 |
|  |  | thiamine(1+) monophosphate | FAD | 1 | 5 | 0.215 | 1zp0(A) | 31.7 | 26 | 0 | 2 | 0 |
| 3.1.1.22 | Q8XZR1 | (R)-3-hydroxybutyric acid | MLA | 6 | 4 | 0.765 | 3v1k(A) | 36.7 | 25 | -6 | 25 | -24 |
|  |  | (R)-3-hydroxybutyrate | MLI | 6 | 3 | 0.778 | 2pu5(A) | 36.7 | 25 | -6 | 32 | -18.75 |
|  |  | (R)-3-[(R)-3-hydroxybutanoyloxy]butanoic acid | MLA | 6 | 4 | 0.289 | 3v1k(A) | 36.7 | 25 | -6 | 25 | -24 |
|  |  | (R)-3-[(R)-3-hydroxybutanoyloxy]butanoate | MLI | 6 | 3 | 0.304 | 2pu5(A) | 36.7 | 25 | -6 | 32 | -18.75 |
| 2.1.1.181 | Q8ZQN4 | S-adenosyl-L-methionine | SAH | 1 | 1 | 0.998 | 2h00(B) | 36.1 | 4.40E-16 | 29 | 43 | 67.442 |
|  |  | S-adenosyl-L-homocysteine | SAH | 1 | 1 | 1 | 2h00(B) | 36.1 | 4.40E-16 | 29 | 43 | 67.442 |
| 1.14.11.8 | Q91ZE0 | 2-oxoglutaric acid | AKG | 2 | 1 | 1 | 1gy9(A) | 19 | 18 | 17 | 28 | 60.714 |
|  |  | succinic acid | AKG | 2 | 1 | 0.692 | 1gy9(A) | 19 | 18 | 17 | 28 | 60.714 |
|  |  | N(6),N(6),N(6)-trimethyl-L-lysine | NM2 | 3 | 1 | 0.568 | 3o2g(A) | 30 | 1.30E-25 | 16 | 21 | 76.19 |
|  |  | 3-hydroxy-N(6),N(6),N(6)-trimethyl-L-lysine | NM2 | 3 | 1 | 0.587 | 3o2g(A) | 30 | 1.30E-25 | 16 | 21 | 76.19 |
| 4.1.2.9 | Q937F6 | phosphoric acid | PO4 | 3 | 1 | 0.857 | 3ahf(A) | 52 | 2.40E-88 | 10 | 10 | 100 |
|  |  | acetyl dihydrogen phosphate | PO4 | 3 | 1 | 0.25 | 3ahf(A) | 52 | 2.40E-88 | 10 | 10 | 100 |
|  |  | D-xylulose 5-phosphate | GOL | 4 | 2 | 0.161 | 3ahf(A) | 42.1 | 2.80E-25 | 1 | 10 | 10 |
|  |  | D-glyceraldehyde 3-phosphate | GOL | 4 | 2 | 0.25 | 3ahf(A) | 42.1 | 2.80E-25 | 1 | 10 | 10 |
| 2.4.1.67 | Q93XK2 | raffinose | SUC-GLA | 3 | 1 | 1 | 3lrm(A) | 25 | 3 | 10 | 12 | 83.333 |
|  |  | stachyose | SUC-GLA | 3 | 1 | 1 | 3lrm(A) | 25 | 3 | 10 | 12 | 83.333 |
|  |  | alpha-D-galactosyl-(1->3)-1D-myo-inositol | SUC-GLA | 3 | 1 | 0.571 | 3lrm(A) | 25 | 3 | 10 | 12 | 83.333 |
|  |  | myo-inositol | ARA | 4 | 3 | 0.333 | 3a22(A) | 25.9 | 5.5 | 7 | 12 | 58.333 |
| 2.7.1.43 | Q93ZC9 | ATP | ADP | 1 | 2 | 0.983 | 2a2c(A) | 33 | 5.3 | 19 | 34 | 55.882 |
|  |  | ADP | ADP | 1 | 2 | 1 | 2a2c(A) | 33 | 5.3 | 19 | 34 | 55.882 |
|  |  | D-glucopyranuronic acid | GLA | 3 | 1 | 0.877 | 2dei(A) | 23.9 | 14 | 14 | 22 | 63.636 |
|  |  | D-glucuronate 1-phosphate | NG1 | 2 | 1 | 0.563 | 2a2c(A) | 33 | 2.2 | 20 | 35 | 57.143 |
| 1.1.2.4 | Q94AX4 | pyruvic acid | ACY | 9 | 2 | 0.571 | 1wvf(A) | 32.4 | 0.31 | 5 | 11 | 45.455 |
|  |  | (R)-lactic acid | ACY | 9 | 2 | 0.615 | 1wvf(A) | 32.4 | 0.31 | 5 | 11 | 45.455 |
| 1.13.11.19 | Q96SZ5 | cysteamine | EDO | 9 | 2 | 0.154 | 2gm6(A) | 32.3 | 0.061 | 6 | 15 | 40 |
|  |  | hypotaurine | SO4 | 4 | 1 | 0.273 | 3uss(A) | 30.5 | 1.2 | 12 | 16 | 75 |
| 1.14.13.30 | Q99N16 | leukotriene B4 | PAM | 15 | 1 | 0.506 | 1izo(A) | 21.7 | 0.14 | 10 | 17 | 58.824 |
|  |  | 20-hydroxy-leukotriene B4 | PAM | 15 | 1 | 0.506 | 1izo(A) | 21.7 | 0.14 | 10 | 17 | 58.824 |
|  |  | NADPH | HEM-KLN | 1 | 5 | 0.575 | 2v0m(A) | 28.2 | 5.60E-28 | 44 | 73 | 60.274 |
|  |  | NADP(+) | HEM-KLN | 1 | 5 | 0.558 | 2v0m(A) | 28.2 | 5.60E-28 | 44 | 73 | 60.274 |
| 1.2.1.68 | Q9A777 | NADPH | NDP | 1 | 3 | 1 | 2wox(A) | 30.5 | 2.10E-10 | 49 | 58 | 84.483 |
|  |  | NADH | NAI | 1 | 5 | 1 | 2imp(A) | 29.3 | 2.50E-07 | 35 | 50 | 70 |
|  |  | NADP(+) | NAP | 1 | 1 | 1 | 1uxr(A) | 24.5 | 9.20E-08 | 53 | 71 | 74.648 |
|  |  | NAD(+) | NAD | 1 | 2 | 0.998 | 4i8p(A) | 29.5 | 2.00E-14 | 51 | 65 | 78.462 |
|  |  | ferulic acid | I3E | 9 | 2 | 0.282 | 3sz9(A) | 34 | 5.30E-13 | 10 | 23 | 43.478 |
|  |  | coniferyl aldehyde | I1E | 9 | 1 | 0.296 | 3szb(A) | 31.3 | 4.40E-21 | 12 | 18 | 66.667 |
| 1.1.1.257 | Q9AHG1 | NADH | NAI | 1 | 5 | 1 | 2j6l(A) | 27.9 | 3.10E-14 | 42 | 59 | 71.186 |
|  |  | NAD(+) | NAD | 1 | 2 | 0.998 | 1o04(A) | 33.3 | 6.50E-27 | 50 | 62 | 80.645 |
|  |  | 4-formylbenzenesulfonic acid | I1E | 9 | 2 | 0.377 | 3szb(A) | 32 | 1.10E-10 | 12 | 18 | 66.667 |
|  |  | 4-(hydroxymethyl)benzenesulfonic acid | I1E | 9 | 2 | 0.299 | 3szb(A) | 32 | 1.10E-10 | 12 | 18 | 66.667 |
| 5.5.1.15 | Q9AJE4 | 2-trans,6-trans,10-trans-geranylgeranyl diphosphate | SQA | 8 | 1 | 0.244 | 1ump(A) | 31.3 | 2.60E-08 | 3 | 24 | 12.5 |
| 6.2.1.7 | Q9ES38 | AMP | AMP | 1 | 9 | 1 | 3cw9(A) | 28.7 | 5.7 | 15 | 32 | 46.875 |
|  |  | ATP | ATP | 1 | 6 | 1 | 4gxq(A) | 30.4 | 0.0036 | 18 | 41 | 43.902 |
|  |  | coenzyme A | COA | 1 | 14 | 1 | 1pg4(A) | 27.4 | 0.052 | 2 | 14 | 14.286 |
|  |  | choloyl-CoA | MCA | 1 | 8 | 0.926 | 3nyq(A) | 31.4 | 0.0044 | 15 | 31 | 48.387 |
|  |  | diphosphoric acid | POP | 17 | 1 | 0.923 | 3lnv(A) | 22.4 | 5.4 | 2 | 10 | 20 |
|  |  | diphosphate(4-) | POP | 17 | 1 | 1 | 3lnv(A) | 22.4 | 5.4 | 2 | 10 | 20 |
|  |  | cholic acid | MQ7 | 23 | 1 | 0.406 | 2vpw(C) | 38.5 | 0.039 | -7 | 15 | -46.667 |
| 1.7.1.6 | Q9FAW5 | NADPH | NAD | 1 | 5 | 0.823 | 1xaj(A) | 22.7 | 45 | 18 | 52 | 34.615 |
|  |  | NADP(+) | NAD | 1 | 5 | 0.919 | 1xaj(A) | 22.7 | 45 | 18 | 52 | 34.615 |
|  |  | N,N-dimethyl-1,4-phenylenediamine | FNR | 1 | 4 | 0.044 | 3u7r(A) | 26.2 | 0.00038 | 18 | 33 | 54.545 |
|  |  | aniline | 2PE | 4 | 1 | 0.032 | 3u7r(A) | 26.2 | 0.00038 | 5 | 12 | 41.667 |
| 2.3.1.75 | Q9FJ75 | coenzyme A | NAG-NAG-BMA-MAN-MAN-FUC | 1 | 1 | 0.427 | 3gmo(A) | 23.1 | 25 | 15 | 23 | 65.217 |
| 2.3.1.166 | Q9FPW3 | coenzyme A | MLC | 1 | 1 | 0.96 | 2e1t(A) | 28.3 | 0.006 | 33 | 49 | 67.347 |
|  |  | benzoyl-CoA | MLC | 1 | 1 | 0.95 | 2e1t(A) | 28.3 | 0.006 | 33 | 49 | 67.347 |
|  |  | 10-deacetylbaccatin III | MLC | 1 | 1 | 0.276 | 2e1t(A) | 28.3 | 0.006 | 33 | 49 | 67.347 |
|  |  | 10-deacetyl-2-debenzoylbaccatin III | MLC | 1 | 1 | 0.244 | 2e1t(A) | 28.3 | 0.006 | 33 | 49 | 67.347 |
| 4.2.3.71 | Q9FQ27 | diphosphoric acid | POP | 2 | 1 | 0.923 | 1n23(A) | 31.4 | 2.40E-34 | 24 | 30 | 80 |
|  |  | diphosphate(4-) | POP | 2 | 1 | 1 | 1n23(A) | 31.4 | 2.40E-34 | 24 | 30 | 80 |
|  |  | 2-trans,6-trans-farnesyl diphosphate | FGG | 5 | 1 | 0.817 | 3p5r(A) | 30 | 6.50E-32 | 21 | 39 | 53.846 |
|  |  | (1E,4E)-germacrene B | 1GA | 12 | 1 | 0.29 | 4di5(A) | 47.6 | 1.00E-66 | 11 | 18 | 61.111 |
| 4.2.1.124 | Q9FR95 | (S)-2,3-epoxysqualene | LAN | 3 | 1 | 0.323 | 1w6k(A) | 39.3 | 3.90E-57 | 18 | 31 | 58.065 |
| 2.4.1.99 | Q9FSV7 | sucrose | SUC | 1 | 1 | 1 | 2qqw(A) | 37.9 | 8.50E-38 | 27 | 32 | 84.375 |
|  |  | 1-kestose | DQR | 1 | 3 | 1 | 3ugg(A) | 58.5 | 3.40E-28 | 24 | 35 | 68.571 |
|  |  | D-glucopyranose | FRU | 3 | 1 | 0.683 | 2xqr(A) | 51.2 | 1.00E-15 | 26 | 32 | 81.25 |
| 3.2.1.51 | Q9FXE5 | L-fucopyranose | GOL | 1 | 1 | 0.093 | 1yht(A) | 21.8 | 3.7 | 5 | 9 | 55.556 |
| 1.14.13.71 | Q9FXW4 | NADPH | ANP | 15 | 1 | 0.752 | 3vpd(A) | 33.3 | 48 | 12 | 23 | 52.174 |
|  |  | NADP(+) | ANP | 15 | 1 | 0.787 | 3vpd(A) | 33.3 | 48 | 12 | 23 | 52.174 |
|  |  | (S)-N-methylcoclaurine | TIC | 26 | 2 | 0.316 | 3kw4(A) | 27.4 | 6.50E-14 | 9 | 22 | 40.909 |
|  |  | (S)-3'-hydroxy-N-methylcoclaurine | TIC | 26 | 2 | 0.314 | 3kw4(A) | 27.4 | 6.50E-14 | 9 | 22 | 40.909 |
| 1.14.12.20 | Q9FYC2 | NADPH | 145 | 5 | 4 | 0.299 | 1jyv(A) | 26.7 | 33 | 5 | 18 | 27.778 |
|  |  | NADP(+) | 145 | 5 | 4 | 0.304 | 1jyv(A) | 26.7 | 33 | 5 | 18 | 27.778 |
| 1.14.11.13 | Q9FZ21 | 2-oxoglutaric acid | AKG | 1 | 1 | 1 | 2brt(A) | 29 | 6.10E-19 | 25 | 25 | 100 |
|  |  | succinic acid | SIN | 1 | 2 | 1 | 1gp6(A) | 29 | 6.10E-19 | 15 | 17 | 88.235 |
|  |  | gibberellin A1 | NAR | 2 | 3 | 0.326 | 2brt(A) | 29 | 6.10E-19 | 8 | 21 | 38.095 |
|  |  | gibberellin A8 | NAR | 2 | 3 | 0.326 | 2brt(A) | 29 | 6.10E-19 | 8 | 21 | 38.095 |
| 2.4.99.7 | Q9H4F1 | CMP | C5P | 1 | 1 | 1 | 2wnb(A) | 29.5 | 5.70E-08 | 15 | 29 | 51.724 |
|  |  | CMP-N-acetyl-beta-neuraminic acid | C5P | 1 | 1 | 0.561 | 2wnb(A) | 29.5 | 5.70E-08 | 15 | 29 | 51.724 |
| 1.13.12.4 | Q9HDX2 | acetic acid | ACY | 6 | 3 | 1 | 1tb3(A) | 35 | 7.90E-22 | 11 | 11 | 100 |
|  |  | (S)-lactic acid | LAC | 6 | 4 | 1 | 2nli(B) | 28.5 | 5.90E-16 | 9 | 9 | 100 |
| 4.1.1.75 | Q9HUI8 | 5-guanidino-2-oxopentanoic acid | GVA | 30 | 1 | 0.831 | 2ihv(A) | 29.9 | 1.70E-08 | 4 | 18 | 22.222 |
|  |  | 4-guanidinobutanal | GVA | 30 | 1 | 0.589 | 2ihv(A) | 29.9 | 1.70E-08 | 4 | 18 | 22.222 |
| 6.6.1.2 | Q9HZQ3 | ATP | FAD | 1 | 1 | 0.687 | 4at0(A) | 26.8 | 0.065 | 24 | 83 | 28.916 |
|  |  | ADP | FAD | 1 | 1 | 0.684 | 4at0(A) | 26.8 | 0.065 | 24 | 83 | 28.916 |
|  |  | phosphoric acid | EDO | 8 | 2 | 0.091 | 3fj1(B) | 27 | 27 | 3 | 7 | 42.857 |
|  |  | cob(II)yrinic acid a,c diamide | FAD | 1 | 1 | 0.439 | 4at0(A) | 26.8 | 0.065 | 24 | 83 | 28.916 |
|  |  | hydrogenobyrinic acid a,c-diamide | FAD | 1 | 1 | 0.407 | 4at0(A) | 26.8 | 0.065 | 24 | 83 | 28.916 |
| 6.2.1.32 | Q9I4X3 | AMP | AMP | 1 | 3 | 1 | 2p2f(A) | 26.9 | 0.0031 | 21 | 38 | 55.263 |
|  |  | ATP | ATP | 1 | 5 | 1 | 3c5e(A) | 27.8 | 0.0008 | 20 | 51 | 39.216 |
|  |  | coenzyme A | COA | 1 | 8 | 1 | 3gpc(A) | 27.8 | 0.0008 | 18 | 41 | 43.902 |
|  |  | anthraniloyl-CoA | BCO | 1 | 12 | 0.944 | 3eq6(A) | 27.5 | 0.00018 | 9 | 25 | 36 |
|  |  | anthranilic acid | BEZ | 2 | 1 | 0.7 | 2v7b(A) | 29.5 | 0.0041 | 16 | 21 | 76.19 |
|  |  | diphosphoric acid | PO4 | 21 | 1 | 0.461 | 3ntl(A) | 28.8 | 34 | 2 | 4 | 50 |
|  |  | diphosphate(4-) | PO4 | 21 | 1 | 0.538 | 3ntl(A) | 28.8 | 34 | 2 | 4 | 50 |
| 2.8.2.11 | Q9JHE4 | adenosine 3',5'-bismonophosphate | PLP | 3 | 1 | 0.166 | 3nzp(A) | 26.1 | 32 | -13 | 35 | -37.143 |
|  |  | 3'-phospho-5'-adenylyl sulfate | PLP | 3 | 1 | 0.163 | 3nzp(A) | 26.1 | 32 | -13 | 35 | -37.143 |
| 2.4.1.224 | Q9JKV7 | UDP | UDP | 1 | 3 | 1 | 1on8(A) | 27.9 | 2.60E-17 | 16 | 37 | 43.243 |
|  |  | UDP-N-acetyl-alpha-D-glucosamine | UD2 | 1 | 1 | 1 | 1omz(A) | 27.3 | 4.20E-17 | 23 | 48 | 47.917 |
| 2.1.1.156 | Q9KJ22 | S-adenosyl-L-methionine | SAM | 1 | 1 | 1 | 1nbi(A) | 36.7 | 1.10E-10 | 35 | 46 | 76.087 |
|  |  | S-adenosyl-L-homocysteine | SAM | 1 | 1 | 1 | 1nbi(A) | 36.7 | 1.10E-10 | 35 | 46 | 76.087 |
|  |  | N,N-dimethylglycine | ACT | 10 | 1 | 0.474 | 3dtn(A) | 32.1 | 0.093 | 8 | 15 | 53.333 |
|  |  | glycine | ACT | 10 | 1 | 0.6 | 3dtn(A) | 32.1 | 0.093 | 8 | 15 | 53.333 |
|  |  | sarcosine | ACT | 10 | 1 | 0.474 | 3dtn(A) | 32.1 | 0.093 | 8 | 15 | 53.333 |
| 1.1.1.312 | Q9KWL3 | NADPH | NDP | 1 | 1 | 1.000 | 1h6a(A) | 32.8 | 1.10E-08 | 31 | 53 | 58.491 |
|  |  | NADP(+) | NAP | 1 | 3 | 0.998 | 1zh8(A) | 27.2 | 0.00029 | 26 | 46 | 56.522 |
| 3.5.99.5 | Q9KWS2 | 2-aminomuconic acid | SER | 2 | 1 | 0.308 | 2uyk(C) | 30.1 | 5.10E-09 | 9 | 9 | 100 |
| 3.5.1.56 | Q9LCC1 | formic acid | ADP | 1 | 1 | 0.01 | 3ax6(A) | 25.2 | 38 | 10 | 24 | 41.667 |
|  |  | formate | ADP | 1 | 1 | 0.01 | 3ax6(A) | 25.2 | 38 | 10 | 24 | 41.667 |
|  |  | N,N-dimethylformamide | ADP | 1 | 1 | 0.016 | 3ax6(A) | 25.2 | 38 | 10 | 24 | 41.667 |
|  |  | dimethylamine | ADP | 1 | 1 | 0.008 | 3ax6(A) | 25.2 | 38 | 10 | 24 | 41.667 |
| 2.4.1.43 | Q9LE59 | UDP | UPF | 1 | 1 | 0.826 | 1ga8(A) | 24.3 | 1.80E-06 | 24 | 61 | 39.344 |
|  |  | UDP-alpha-D-galacturonic acid | UPF | 1 | 1 | 0.809 | 1ga8(A) | 24.3 | 1.80E-06 | 24 | 61 | 39.344 |
| 2.1.1.128 | Q9LEL6 | S-adenosyl-L-methionine | SAM | 1 | 2 | 1 | 3i5u(A) | 26.7 | 2.10E-09 | 32 | 42 | 76.19 |
|  |  | S-adenosyl-L-homocysteine | SAH | 1 | 1 | 1 | 3reo(A) | 40.5 | 3.70E-40 | 38 | 42 | 90.476 |
|  |  | (RS)-coclaurine | ASE | 4 | 1 | 0.359 | 4a6e(A) | 23.8 | 1.40E-14 | 15 | 28 | 53.571 |
|  |  | (RS)-norcoclaurine | ASE | 4 | 1 | 0.383 | 4a6e(A) | 23.8 | 1.40E-14 | 15 | 28 | 53.571 |
| 3.2.1.167 | Q9LRC8 | D-glucopyranuronic acid | NAG | 14 | 1 | 0.456 | 3hrz(A) | 27.3 | 26 | 3 | 8 | 37.5 |
|  |  | baicalein | NAG-NAG-BMA-BMA-MAN-MAN-MAN-MAN-MAN | 7 | 5 | 0.278 | 1tg7(A) | 20.5 | 4.9 | -18 | 49 | -36.735 |
|  |  | baicalin | NAG-NAG-BMA-BMA-MAN-MAN-MAN-MAN-MAN | 7 | 5 | 0.472 | 1tg7(A) | 20.5 | 4.9 | -18 | 49 | -36.735 |
| 2.5.1.45 | Q9M4B0 | spermidine | GC7 | 2 | 1 | 0.35 | 1rqd(A) | 62.1 | 4.70E-99 | 22 | 23 | 95.652 |
|  |  | sym-homospermidine | GC7 | 2 | 1 | 0.368 | 1rqd(A) | 62.1 | 4.70E-99 | 22 | 23 | 95.652 |
|  |  | putrescine | GC7 | 2 | 1 | 0.345 | 1rqd(A) | 62.1 | 4.70E-99 | 22 | 23 | 95.652 |
|  |  | trimethylenediamine | GC7 | 2 | 1 | 0.233 | 1rqd(A) | 62.1 | 4.70E-99 | 22 | 23 | 95.652 |
| 5.5.1.9 | Q9M643 | cycloeucalenol | ATP | 1 | 1 | 0.12 | 2aqx(A) | 48 | 6.2 | 9 | 45 | 20 |
|  |  | obtusifoliol | ATP | 1 | 1 | 0.13 | 2aqx(A) | 48 | 6.2 | 9 | 45 | 20 |
| 1.14.13.118 | Q9M7B7 | NADPH | HEM-POZ | 1 | 23 | 0.573 | 3k1o(A) | 19.4 | 4.10E-05 | 28 | 67 | 41.791 |
|  |  | NADP(+) | HEM-POZ | 1 | 23 | 0.552 | 3k1o(A) | 19.4 | 4.10E-05 | 28 | 67 | 41.791 |
|  |  | L-valine | TAM | 46 | 1 | 0.52 | 3uas(A) | 22.2 | 0.0011 | 5 | 13 | 38.462 |
|  |  | 2-methylpropanal oxime | TAM | 46 | 1 | 0.296 | 3uas(A) | 22.2 | 0.0011 | 5 | 13 | 38.462 |
| 2.4.1.210 | Q9MB73 | UDP | UDP | 1 | 2 | 1 | 2vch(A) | 20.2 | 47 | 33 | 41 | 80.488 |
|  |  | UDP-D-glucose | UPG | 1 | 3 | 0.855 | 2acw(A) | 37.5 | 2.60E-20 | 30 | 44 | 68.182 |
|  |  | UDP-alpha-D-glucose | UPG | 1 | 3 | 0.855 | 2acw(A) | 37.5 | 2.60E-20 | 30 | 44 | 68.182 |
|  |  | limonin | ZIO | 2 | 1 | 0.415 | 2iya(A) | 27 | 0.0088 | 23 | 45 | 51.111 |
|  |  | limonin 17-beta-D-glucoside | ZIO | 2 | 1 | 0.477 | 2iya(A) | 27 | 0.0088 | 23 | 45 | 51.111 |
| 4.1.1.90 | Q9MYY3 | phylloquinone | HEM | 12 | 1 | 0.268 | 2f9q(A) | 38.6 | 1.9 | 3 | 15 | 20 |
|  |  | vitamin K epoxide | FAD | 1 | 1 | 0.264 | 3vte(A) | 21.3 | 2 | 18 | 71 | 25.352 |
| 1.1.1.300 | Q9NYR8 | NADPH | NDP | 1 | 2 | 1 | 2jap(A) | 33.7 | 3.00E-15 | 57 | 68 | 83.824 |
|  |  | NADP(+) | NAP | 1 | 1 | 0.998 | 3hb5(X) | 46.9 | 3.10E-48 | 61 | 73 | 83.562 |
|  |  | all-trans-retinol | EST | 2 | 1 | 0.44 | 1fdu(A) | 46.9 | 5.00E-48 | 22 | 28 | 78.571 |
|  |  | all-trans-retinal | EST | 2 | 1 | 0.434 | 1fdu(A) | 46.9 | 5.00E-48 | 22 | 28 | 78.571 |
| 2.1.1.109 | Q9P900 | S-adenosyl-L-methionine | SAM | 1 | 2 | 1 | 4a6e(A) | 30.7 | 1.60E-09 | 26 | 42 | 61.905 |
|  |  | S-adenosyl-L-homocysteine | SAH | 1 | 1 | 1 | 3tky(A) | 27.2 | 1.10E-10 | 29 | 40 | 72.5 |
|  |  | sterigmatocystin | HMK | 10 | 2 | 0.453 | 1zga(A) | 32.7 | 5.70E-10 | 5 | 25 | 20 |
|  |  | 6-demethylsterigmatocystin | HMK | 10 | 2 | 0.461 | 1zga(A) | 32.7 | 5.70E-10 | 5 | 25 | 20 |
| 1.1.1.170 | Q9R1J0 | NADPH | NDP | 1 | 5 | 1 | 2gn4(A) | 24.3 | 0.017 | 28 | 74 | 37.838 |
|  |  | NADH | NAI | 1 | 4 | 1 | 1ek6(A) | 30.1 | 1.7 | 35 | 62 | 56.452 |
|  |  | NADP(+) | NAP | 1 | 2 | 0.998 | 2nnl(D) | 29.8 | 8.5 | 38 | 62 | 61.29 |
|  |  | NAD(+) | NAD | 1 | 1 | 0.998 | 3icp(A) | 31.6 | 2.20E-09 | 44 | 78 | 56.41 |
| 1.21.4.2 | Q9R4G7 | acetyl dihydrogen phosphate | SO4 | 1 | 1 | 0.031 | 2d62(A) | 25 | 3.1 | 5 | 13 | 38.462 |
|  |  | glycine | SO4 | 1 | 1 | 0.048 | 2d62(A) | 25 | 3.1 | 5 | 13 | 38.462 |
|  |  | phosphoric acid | SO4 | 1 | 1 | 0.077 | 2d62(A) | 25 | 3.1 | 5 | 13 | 38.462 |
| 2.1.1.101 | Q9S4D5 | S-adenosyl-L-methionine | SAH | 1 | 1 | 0.998 | 2wk1(A) | 52.1 | 1.20E-27 | 47 | 51 | 92.157 |
|  |  | S-adenosyl-L-homocysteine | SAH | 1 | 1 | 1 | 2wk1(A) | 52.1 | 1.20E-27 | 47 | 51 | 92.157 |
|  |  | tylosin | DRA | 5 | 1 | 0.626 | 1xds(A) | 31 | 19 | 3 | 21 | 14.286 |
|  |  | macrocin | DRA | 5 | 1 | 0.626 | 1xds(A) | 31 | 19 | 3 | 21 | 14.286 |
| 2.5.1.79 | Q9S7X6 | 5'-S-methyl-5'-thioadenosine | MTA | 1 | 4 | 1 | 3b7p(A) | 29.6 | 0.065 | 36 | 49 | 73.469 |
|  |  | S-adenosylmethioninamine | S4M | 1 | 1 | 1 | 2pt6(A) | 29.6 | 0.065 | 42 | 54 | 77.778 |
|  |  | spermidine | SPD | 3 | 2 | 1 | 2pwp(A) | 29.6 | 0.065 | 19 | 27 | 70.37 |
| 4.2.3.26 | Q9SPN1 | diphosphoric acid | POP | 4 | 1 | 0.923 | 1n23(A) | 40.2 | 4.80E-54 | 30 | 30 | 100 |
|  |  | diphosphate(4-) | POP | 4 | 1 | 1 | 1n23(A) | 40.2 | 4.80E-54 | 30 | 30 | 100 |
|  |  | geranyl diphosphate | FGG | 6 | 1 | 0.742 | 3p5r(A) | 27.9 | 3.80E-29 | 22 | 39 | 56.41 |
|  |  | (R)-linalool | FHP | 9 | 1 | 0.284 | 1hxa(A) | 30.2 | 7.10E-35 | 18 | 33 | 54.545 |
| 1.3.7.4 | Q9SR43 | (3Z)-phytochromobilin | BLA | 1 | 1 | 0.957 | 2x9o(A) | 25.2 | 2.60E-08 | 24 | 47 | 51.064 |
| 2.6.1.80 | Q9ST02 | 2-oxoglutaric acid | AKG | 10 | 1 | 1 | 3ath(A) | 26.8 | 7.50E-09 | 14 | 27 | 51.852 |
|  |  | L-glutamic acid | GLU | 12 | 1 | 1 | 1gde(A) | 26.8 | 1.4 | 9 | 13 | 69.231 |
|  |  | (S,S,S)-nicotianamine | PGU | 3 | 3 | 0.236 | 3ei5(A) | 24.1 | 9.10E-07 | 28 | 34 | 82.353 |
| 4.2.3.40 | Q9T0J9 | diphosphoric acid | POP | 2 | 1 | 0.923 | 1n23(A) | 25.2 | 4.40E-18 | 25 | 30 | 83.333 |
|  |  | diphosphate(4-) | POP | 2 | 1 | 1 | 1n23(A) | 25.2 | 4.40E-18 | 25 | 30 | 83.333 |
|  |  | 2-trans,6-trans-farnesyl diphosphate | FGG | 6 | 2 | 0.821 | 3p5r(A) | 24.4 | 1.40E-16 | 17 | 39 | 43.59 |
| 2.4.1.145 | Q9UM21 | UDP | F43 | 3 | 1 | 0.425 | 1e6y(A) | 23.1 | 12 | 9 | 20 | 45 |
|  |  | UDP-N-acetyl-alpha-D-glucosamine | F43 | 3 | 1 | 0.536 | 1e6y(A) | 23.1 | 12 | 9 | 20 | 45 |
| 2.1.1.203 | Q9W4M9 | S-adenosyl-L-methionine | SAM | 1 | 1 | 1 | 4fzv(A) | 30.3 | 0.058 | 42 | 51 | 82.353 |
|  |  | S-adenosyl-L-homocysteine | SAM | 1 | 1 | 0.998 | 4fzv(A) | 30.3 | 0.058 | 42 | 51 | 82.353 |
| 4.2.3.21 | Q9XJ32 | diphosphoric acid | POP | 3 | 1 | 0.923 | 1n23(A) | 32.2 | 3.40E-42 | 28 | 30 | 93.333 |
|  |  | diphosphate(4-) | POP | 3 | 1 | 1 | 1n23(A) | 32.2 | 3.40E-42 | 28 | 30 | 93.333 |
|  |  | 2-trans,6-trans-farnesyl diphosphate | FGG | 2 | 4 | 0.817 | 3p5r(A) | 28.9 | 1.60E-28 | 20 | 39 | 51.282 |
|  |  | premnaspirodiene | 7A8 | 14 | 1 | 0.298 | 1n22(A) | 32.2 | 3.40E-42 | 9 | 18 | 50 |
| 2.8.2.29 | Q9Y278 | 3'-phospho-5'-adenylyl sulfate | A3P | 1 | 1 | 0.953 | 1t8u(B) | 74 | 1.90E-95 | 47 | 47 | 100 |
| 2.1.1.86 | Q9Y8K6 | 5,6,7,8-tetrahydromethanopterin | NAP | 1 | 2 | 0.489 | 1nvt(A) | 35.2 | 4.3 | 2 | 7 | 28.571 |
|  |  | coenzyme M | NAP | 1 | 2 | 0.017 | 1nvt(A) | 35.2 | 4.3 | 2 | 7 | 28.571 |
|  |  | methyl-CoM | NAP | 1 | 2 | 0.023 | 1nvt(A) | 35.2 | 4.3 | 2 | 7 | 28.571 |
| 2.1.1.102 | Q9ZHQ4 | S-adenosyl-L-methionine | SAH | 1 | 1 | 0.998 | 3ssn(C) | 56 | 3.50E-40 | 49 | 50 | 98 |
|  |  | S-adenosyl-L-homocysteine | SAH | 1 | 1 | 1.000 | 3ssn(C) | 56 | 3.50E-40 | 49 | 50 | 98 |
|  |  | macrocin | MVI | 2 | 1 | 0.771 | 3ssn(C) | 56 | 3.50E-40 | 27 | 28 | 96.429 |
|  |  | demethylmacrocin | MVI | 2 | 1 | 0.776 | 3ssn(C) | 56 | 3.50E-40 | 27 | 28 | 96.429 |
| 2.3.1.107 | Q9ZTK5 | coenzyme A | COA | 1 | 2 | 1 | 2zba(A) | 21.1 | 14 | 21 | 40 | 52.5 |
|  |  | acetyl-CoA | MLC | 1 | 1 | 0.983 | 2e1t(A) | 24.7 | 6.6 | 26 | 49 | 53.061 |
|  |  | vindoline | MLC | 1 | 1 | 0.421 | 2e1t(A) | 24.7 | 6.6 | 26 | 49 | 53.061 |
